# The progression of disorder-specific brain pattern expression in schizophrenia over 9 years

**DOI:** 10.1038/s41537-021-00157-0

**Published:** 2021-06-14

**Authors:** Johannes Lieslehto, Erika Jääskeläinen, Vesa Kiviniemi, Marianne Haapea, Peter B. Jones, Graham K. Murray, Juha Veijola, Udo Dannlowski, Dominik Grotegerd, Susanne Meinert, Tim Hahn, Anne Ruef, Matti Isohanni, Peter Falkai, Jouko Miettunen, Dominic B. Dwyer, Nikolaos Koutsouleris

**Affiliations:** 1 Center for Life Course Health Research, University of Oulu, Oulu, Finland; 2 Department of Psychiatry and Psychotherapy, Ludwig-Maximilian-University, Munich, Germany; 3 Department of Forensic Psychiatry, University of Eastern Finland, Niuvanniemi Hospital, Kuopio, Finland; 4 Department of Psychiatry, Oulu University Hospital, Oulu, Finland; 5 Medical Research Center Oulu, Oulu University Hospital and University of Oulu, Oulu, Finland; 6 Department of Diagnostic Radiology, Oulu University Hospital, Oulu, Finland; 7 Department of Psychiatry, University of Cambridge, Cambridge, UK; 8 Department of Psychiatry, Research Unit of Clinical Neuroscience, University of Oulu, Oulu, Finland; 9 Institute for Translational Psychiatry, University of Münster, Münster, Germany; 10 International Max Planck Research School for Translational Psychiatry, Munich, Germany; 11 Institute of Psychiatry, Psychology and Neuroscience, King’s College London, London, UK

**Keywords:** Schizophrenia, Biomarkers

## Abstract

Age plays a crucial role in the performance of schizophrenia vs. controls (SZ-HC) neuroimaging-based machine learning (ML) models as the accuracy of identifying first-episode psychosis from controls is poor compared to chronic patients. Resolving whether this finding reflects longitudinal progression in a disorder-specific brain pattern or a systematic but non-disorder-specific deviation from a normal brain aging (BA) trajectory in schizophrenia would help the clinical translation of diagnostic ML models. We trained two ML models on structural MRI data: an SZ-HC model based on 70 schizophrenia patients and 74 controls and a BA model (based on 561 healthy individuals, age range = 66 years). We then investigated the two models’ predictions in the naturalistic longitudinal Northern Finland Birth Cohort 1966 (NFBC1966) following 29 schizophrenia and 61 controls for nine years. The SZ-HC model’s schizophrenia-specificity was further assessed by utilizing independent validation (62 schizophrenia, 95 controls) and depression samples (203 depression, 203 controls). We found better performance at the NFBC1966 follow-up (sensitivity = 75.9%, specificity = 83.6%) compared to the baseline (sensitivity = 58.6%, specificity = 86.9%). This finding resulted from progression in disorder-specific pattern expression in schizophrenia and was not explained by concomitant acceleration of brain aging. The disorder-specific pattern’s progression reflected longitudinal changes in cognition, outcomes, and local brain changes, while BA captured treatment-related and global brain alterations. The SZ-HC model was also generalizable to independent schizophrenia validation samples but classified depression as control subjects. Our research underlines the importance of taking account of longitudinal progression in a disorder-specific pattern in schizophrenia when developing ML classifiers for different age groups.

## Introduction

Neuroimaging-based machine learning techniques have the potential to improve the accuracy of diagnosing schizophrenia in clinical care^1^. However, previous cross-sectional research shows that the stage of schizophrenia plays a key role in the performance of machine learning classifiers. The accuracy of identifying first-episode psychosis from controls is poor^2^ compared to older and more chronic patients^3^. Given the widely-reported findings of more significant progression of longitudinal structural brain alterations in schizophrenia compared to controls^4–6^, it is possible that the above finding reflects progression in the multivariate patterns that could have multiple underlying sources.

One possibility is that the neuroanatomical pattern distinguishing schizophrenia from controls (SZ-HC) becomes more discernible over time due to the disorder’s progressive nature. This possibility should be explored in a longitudinal sample since the above age-related finding might also reflect selection bias, where those schizophrenia patients meeting recovery in the future (about 13.5%^7^) are missing from chronic samples^8^. This is an important consideration since schizophrenia patients with poor outcomes have more structural abnormalities than those with good outcomes^9,10^.

Another possibility is that the above age-related finding may reflect deviations from a normal brain aging trajectory in schizophrenia that are non-disorder-specific. Brain aging is a synchronized process among individuals, which has enabled the employment of machine learning to estimate chronological age from an individual’s brain with high accuracy^11,12^. In schizophrenia, there appears to be a gradient of aggravation in higher predicted age (vs. chronological age) from at-risk individuals to established diagnosis^13^. Nonetheless, increased brain aging (i.e., predicted - chronological age) is not schizophrenia-specific as it can also be traced in other disorders like depression^11,13,14^. Furthermore, previous work has shown that the patient status can be accurately predicted from controls using only non-disorder-specific brain aging in depression and bipolar disorder in addition to schizophrenia^13^. Therefore, it is possible that brain aging in schizophrenia accommodates the aggregations of secondary effects (e.g., cumulative exposure to antipsychotics) as brain aging is generally considered a proxy of overall brain health^15,16^. Given the cross-sectional report of an association between an individual’s schizophrenia fingerprint expression and brain aging^13^, longitudinal investigations on the two patterns’ trajectories are crucial.

To our knowledge, only one previous study has explored the longitudinal course of these patterns^17^. In the study, schizophrenia patients demonstrated an acceleration of brain aging compared to controls over the follow-up. The schizophrenia-related pattern expression in schizophrenia was also magnified over time, but the change was not significant. These findings might imply that non-disorder-specific abnormalities mainly drive the increment in the distinction of schizophrenia from controls over time. To evaluate this possibility, however, a cross-disorder assessment of different clinical manifestations should also be performed. Furthermore, although some of the schizophrenia patients in the study^17^ were followed up to 13 years, the comparison between schizophrenia and controls was limited only to 3.5 years, limiting the conclusions on these patterns’ longitudinal trajectories. Also, since the magnitude of the aging effect on brain structures varies between different age groups^18^, these investigations should be conducted in a sample with a minimum and, most importantly, harmonized age range. Lastly, to avoid potential selection bias, the longitudinal comparison should ideally be performed in individuals recruited and followed within an epidemiological and naturalistic framework.

We aimed to investigate the expression of the SZ-HC and brain age-related patterns over a nine-year follow-up period to resolve whether the age-related increment in the separability of schizophrenia from controls results from progressive schizophrenia-specific alterations in multivariate brain patterns. To avoid potential selection bias in study sample recruitment^8,19^ and limit the potential variance in aging effect on brain structures across different age groups^18^, we investigated these patterns’ trajectories in a prospective birth cohort with a naturalistic setting. Given the previous cross-sectional machine learning findings of higher sensitivity in older (vs. younger) schizophrenia^3^, we hypothesized that we would find a more accurate classification of schizophrenia patients at the follow-up (vs. the baseline). Second, building upon the existing evidence on schizophrenia’s separability from other psychiatric disorders^20,21^, we hypothesized the SZ-HC classifier is disorder-specific (i.e., the model is not generalizable to a different clinical manifestation). Consequently, we hypothesized that the SZ-HC model and non-disorder-specific brain aging are differently reflected in longitudinal changes in brain structures and clinical variables.

## Results

### Sociodemographic and clinical characteristics

Demographic data are described in the Table 1 and a more detailed description is provided in the Supplement. The schizophrenia patients of the Centre for Biomedical Research Excellence (COBRE) sample were older than the schizophrenia patients of the Northern Finland Birth Cohort 1966 (NFBC1966) at the baseline (Cohen’s d = 0.35, *t*(70) = 2.5, *P* value = 0.01). The Follow-up NFBC1966 schizophrenia patients were older than the COBRE schizophrenia patients (Cohen’s d = 0.42, *t*(70) = −2.9806, *P* value = 0.004). There were more females in the NFBC1966 schizophrenia sample compared to the respective COBRE sample (*χ*^2^ = 8.08, *P* value = 0.004). Schizophrenia onset age did not differ between the COBRE and NFBC1966 (Cohen’s d = 0.26, *t*(85) = −1.4, *P* value = 0.15). There were no Chlorpromazine dose differences between the COBRE and the baseline NFBC1966 (Cohen’s d = 0.02, *t*(41) = −0.07, *P* value = 0.95), and between the COBRE and the follow-up NFBC1966 (Cohen’s d = 0.04, *t*(45) = 0.14, *P* value = 0.89). No differences were found in symptomatic remission between the COBRE and NFBC1966 at baseline (*χ*^2^ = 0.13, *P* value = 0.72), or at follow-up (*χ*^2^ = 0.47, *P* value = 0.49). The number of hospitalizations did not differ between the COBRE and NFBC1966 at baseline (Wilcox U-test:, W = 1124.5, *P* value = 0.22), but there were more hospitalizations at follow-up in NFBC1966 compared with COBRE (Wilcox U-test: W = 1304, *P* value = 0.008).

**Table 1:** Sociodemographic characteristics of the included schizophrenia vs. controls samples.

	COBRE (SZ, *N* = 70)	NFBC1966 (SZ, *N* = 29) *BL/FU*	NMorphCH (SZ, *N* = 44)	CNP (SZ, *N* = 18)	COBRE (HC, *N* = 74)	NFBC1966 (HC, *N* = 61) *BL/FU*	NMorphCH (HC, *N* = 43)	CNP (HC, *N* = 52)
Age [Mean(SD)]	37.83 (13.84)	33.7 (0.75)/42.8 (0.57)	32.45 (6.91)	36.78 (8.75)	35.82 (11.58)	34.5 (0.71)/43.0 (0.54)	31.53 (8.43)	30.71 (9.13)
Sex [Females(%)]	14 (20%)	14 (48%)	14 (32%)	3 (17%)	23 (31%)	23 (38%)	23 (51%)	26 (50%)
School years [Mean(SD)]	12.95 (1.83)	NA	12.79 (1.82)	12.83 (1.62)	14.43 (3.32)	NA	15.96 (2.55)	15.08 (1.75)
Age of illness onset [Mean(SD)]	21.16 (7.56)	22.9 (4.5)	20.65 (4.61)	NA				
Number of hospitalizations [Median(range)]	3 (0–30)	7 (0–31)/11 (0–36)	NA	NA				
Disorder duration [Mean(SD)]	16.49 (12.75)	10.72 (4.5)/19.83 (4.58)	12.35 (7.26)	NA				
Remission [*N* (%)]	20 (29%)	7 (25%)/6 (21%)	2 (6%) NA	3 (17%)				
Positive symptoms [Mean(SD)]	14.73 (4.7)	13.15 (4.88)/15.61 (5.83)	43.71 (33.43)^*^	29.33 (23.73)^*^				
Negative symptoms [Mean(SD)]	14.67 (4.78)	17.44 (10.32)/22.36 (11.39)	58.35 (24.58)^*^	31.44 (16.47)^*^				
CPZ equivalent in mgs [Mean(SD)]	365.84 (307.34)	371.59 (423.7)/354.63 (367.68)	NA	330 (176.18)				

*Assessed with SAPS or SANS.

Remission was assessed according to the Andreasen et al., 2005 without the duration criteria.

*SZ* schizophrenia, *HC* controls, *CPZ* chlorpromazine, *NA* not available, *BL* baseline, *FU* follow-up.

### Schizophrenia classification

**Fig. 1:**
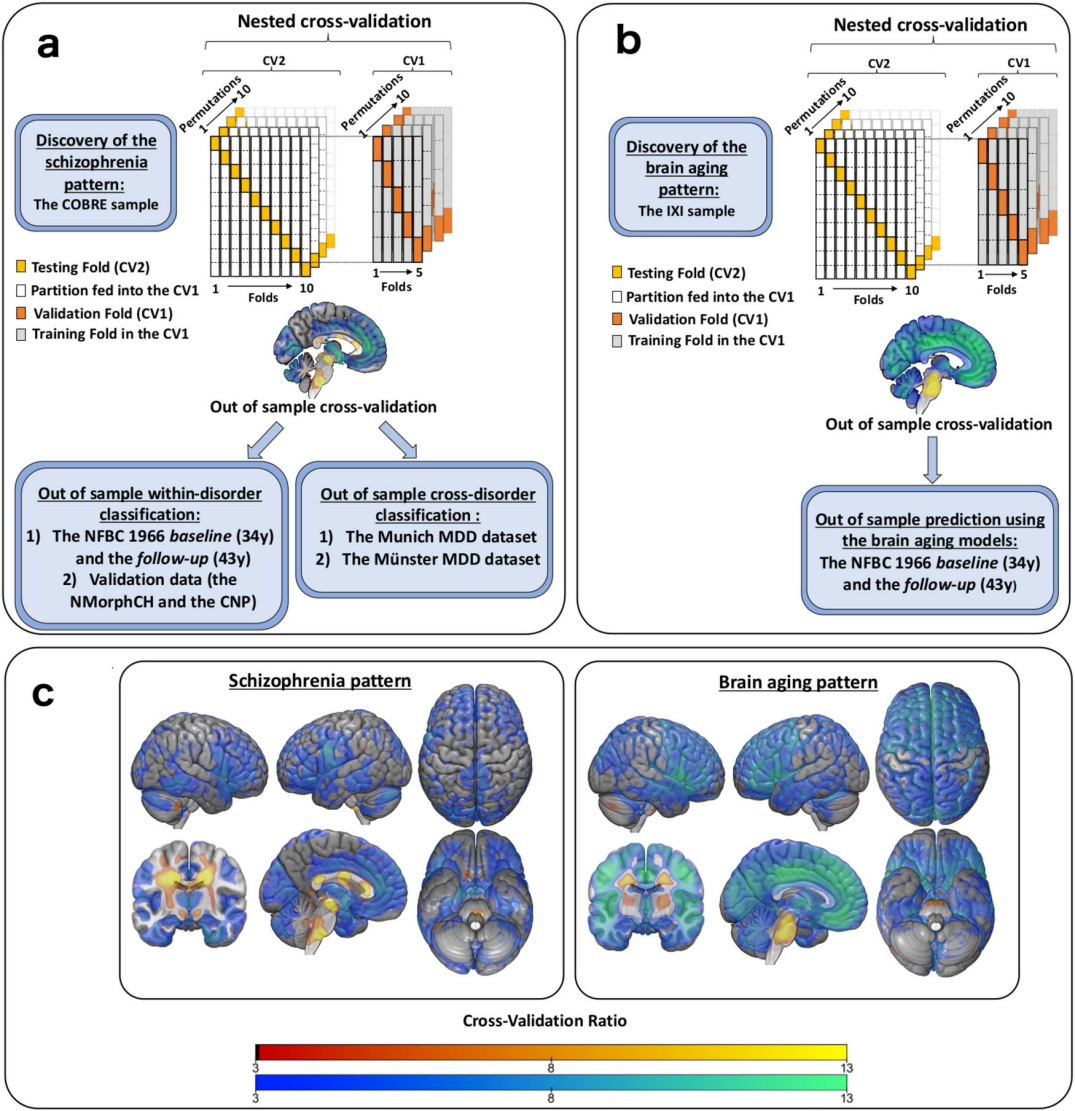
Flowchart depicting the analyses of the study. **a** Training the schizophrenia vs. controls classifier in the COBRE. The classifier was applied, without any in-between retraining, to schizophrenia samples to assess within-disorder and MDD samples to assess cross-disorder performance. **b** Training the brain aging regressor in the IXI. The models were then applied to the NFBC1966 without any in-between retraining. **c** The visualization of the schizophrenia vs. controls classifier and brain aging regressor as cross-validation ratio maps. Warm colours indicate signal increases, and cold colours signal decreases in VBM data.

Classification of schizophrenia from controls in the COBRE model discovery sample resulted in a balanced accuracy (BAC) of 70.8% (sensitivity = 70.0%, specificity = 71.6%). The SVM classifier’s diagnostic pattern’s reliable parts were computed using the cross-validation ratio method of NeuroMiner and shown in (Fig. 1c).

**Fig. 2:**
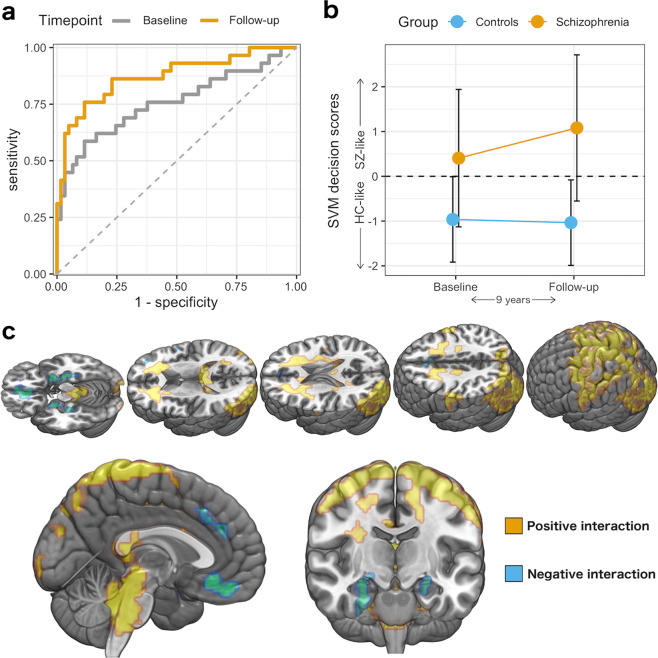
The classification of schizophrenia from controls over time. **a** ROC-curves for the schizophrenia vs. controls classification in the baseline and the follow-up. **b** SVM decision scores over time. Error-bars as standard deviation. SZ schizophrenia, HC healthy controls. **c** The brain regions contributing to the increment in SVM decision scores over time (Threshold-Free Cluster Enhancement-corrected *P* value < 0.05).

Figure 2 presents the ROC curves and SVM decision scores for the SZ-HC classification in NFBC1966 (using the COBRE-trained models). We measured an out-of-sample cross-validation BAC (BAC_oocv_) of 72.8% (sensitivity = 58.6%, specificity = 86.9%) at baseline, whereas BAC_oocv_ measured 79.7% (sensitivity = 75.9%, specificity = 83.6%) at follow-up. Compared to the baseline ROC-curve, the diagnostic separability was significantly better at the follow-up (paired DeLong’s test of two ROC-curves, *Z* = 2.6, *P* value = 0.01). We found an increment in the SVM decision scores over time in the NFBC1966 schizophrenia patients (Cohen’s d = 0.58, *t*(28) = 3.11, *P* value = 0.004) but not in controls (Cohen’s d = 0.09, *t*(60) = −0.7, *P* value = 0.49). We also found an SVM decision score by timepoint interaction (*F*(1,88) = 12.5, *P* value = 0.0007). The interaction was present even when we used BrainAGE as a covariate in the model (*F*(1,89) = 10.5, *P* value = 0.002). There was no sex by group interaction on the SVM decision scores at the baseline (*F*(1,0.28) = 0.189, *P* value = 0.665) or at the follow-up (*F*(1,0.28) = 0.189, *P* value = 0.665).

### Schizophrenia classification in the validation samples

Applying the COBRE-trained classifier to the external validation samples, we measured classification performances of BAC_oocv_ = 77.5% (Sensitivity = 72.2%, Specificity = 82.7%) in the CNP, BAC_oocv_ = 69.1% (Sensitivity = 54.5%, Specificity = 83.7%) in the NMorphCH. The ROC-curves are provided in Supplementary Fig. 5.

### The Performance of the Schizophrenia classifier in classification of MDD from controls

The SZ-HC model’s classification performance in the two MDD datasets resulted in BAC_oocv_ of 61.5% in the Münster (Sensitivity = 35%, Specificity = 88%) and BAC_oocv_ of 49% in the Munich dataset (Sensitivity = 18.4%, Specificity = 79.6%). The SZ-HC classifier’s performance was superior in the external SZ datasets compared to the Münster (DeLong’s test: D = 3.9, *P* value = 0.0001) and the Munich MDD cohorts (D = 6.1, *P* value < 0.0001). Detailed descriptions of the classification performances are provided in the Supplement.

### Brain aging

**Fig. 3:**
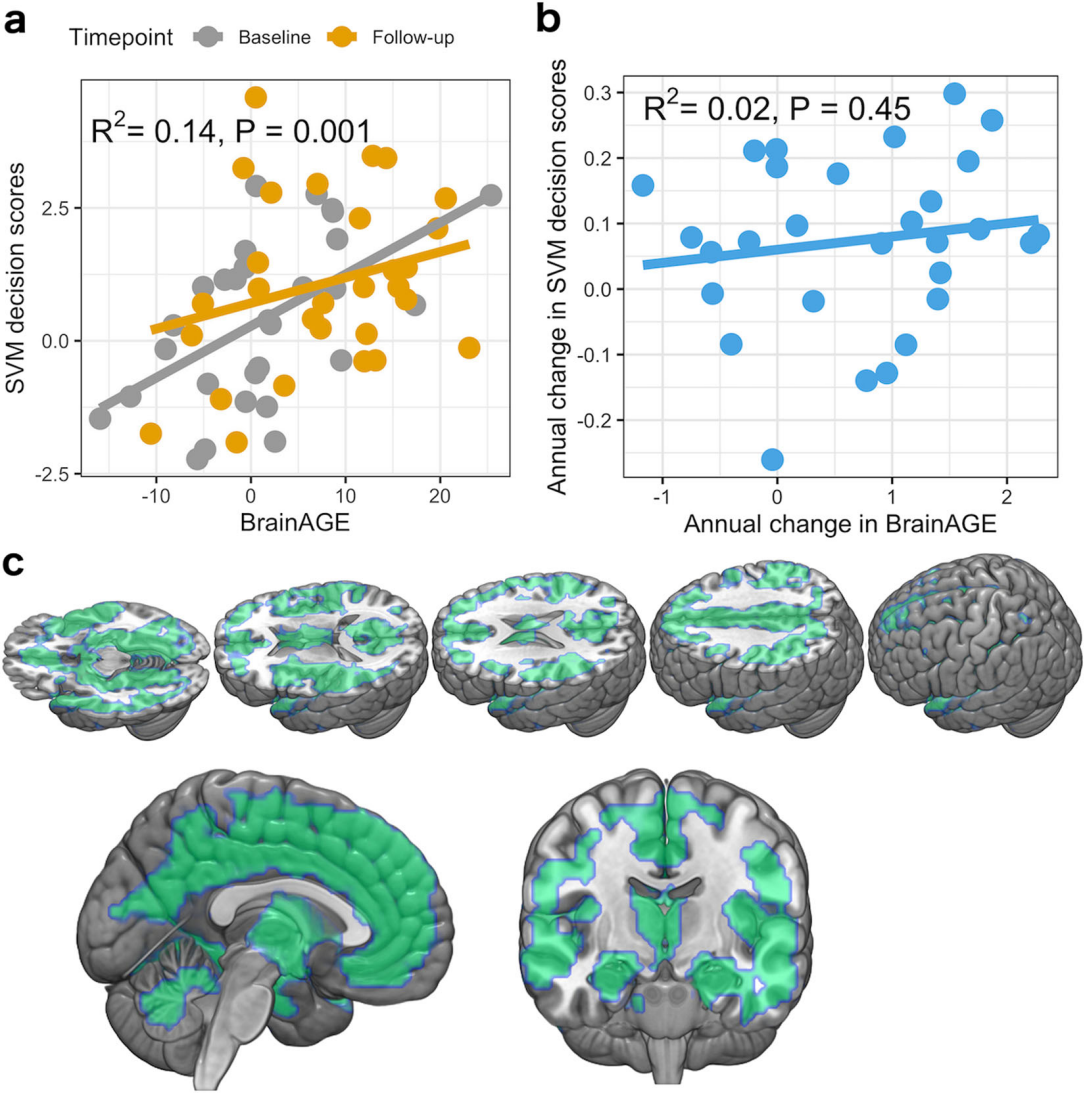
The relation of the SZ-HC model and brain aging over time. **a** The relationship between SVM decision scores and BrainAGE as a coefficient of determination from a linear mixed model. **b** The relationship between the annual change in BrainAGE and annual change in SVM decision scores. **c** The brain regions of which atrophy over time contributes to the corresponding increment in BrainAGE (Threshold-Free Cluster Enhancement-corrected *P* value < 0.05).

Compared to controls, we found higher BrainAGE in patients with schizophrenia at the NFBC1966 baseline (+1.3 years in schizophrenia, *t*(46) = 3.0, Cohen’s d = 0.7, *P* value = 0.005), which was more pronounced at the follow-up (+7.7 years in schizophrenia, *t*(62) = 4.4, Cohen’s d = 0.95, *P* value < 0.0001), which is shown in Supplementary Fig. 9. We also detected a group by timepoint interaction on BrainAGE (*F*(1,88) = 4.0017, *P* value = 0.049). The BrainAGE predicted SVM decision scores for schizophrenia over the follow-up (Fig. 3a) in schizophrenia. However, annual increases in brainAGE did not relate to annual SVM decision score change between the timepoints (Fig. 3b). Across the brain, CV-ratios of the SZ-HC classifier correlated with the brain aging regressor (*ρ* = 0.35, *P* < 0.0001, Supplementary Fig. 15).

### The prediction of patterns’ expressions trajectory with clinical variables

**Fig. 4:**
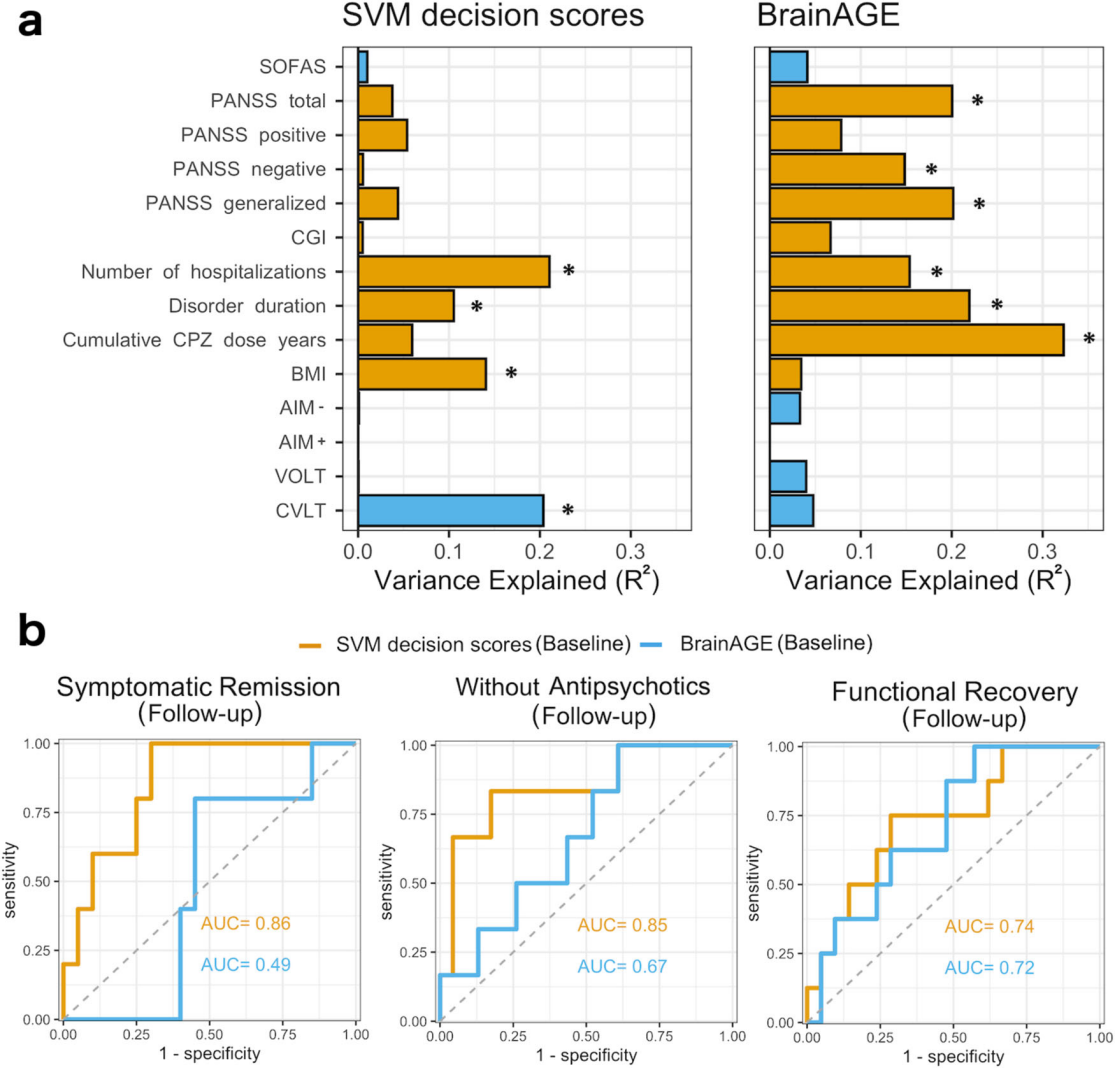
The relation of the two neuroanatomical patterns and clinical and outcome variables. **a** The relationships between the two neuroanatomical patterns’ expressions and clinical variables as coefficients of determinations from linear mixed models. Yellow presents positive and blue presents a negative association. The asterisk presents FDR-corrected *P* value < 0.05. CGI global clinical impression, CPZ chlorpromazine equivalent, AIM- Abstraction, Inhibition and Working Memory without memory part, AIM+ Abstraction, Inhibition and Working Memory with memory part, VOLT Visual Objective Learning Test, CVLT California Verbal Learning Test, BMI body mass index. **b** The performance of baseline SVM decision scores and BrainAGE to predict the follow-up outcomes using the NFBC 1966 schizophrenia (*N* = 29).

Figure 4a, Supplementary Tables 2-3, and Supplementary Figs. 10–11 presents the relationships between the clinical variables with the SVM decision scores and BrainAGE. Across the two timepoints, SVM decision scores had a positive relationship with the number of hospitalizations, disorder duration, and Chlorpromazine dose years, and negatively with the CVLT performance. Of these relationships, only the associations between CVLT and SVM decision scores and BMI and SVM decision scores remained significant after adjusting the model for BrainAGE effects. Post hoc regression analyses revealed the annual decrease in CVLT and increased BMI related to increased SVM decision scores.

Across the two timepoints, total symptoms, negative symptoms, general symptoms, number of hospitalizations, disorder duration, and CPZ dose years had a positive relationship with BrainAGE. These relationships remained significant even when we adjusted the models with SVM decision scores. However, we found that these clinical factors’ annual changes did not relate to annual changes in BrainAGE.

### The prediction of future outcomes using baseline patterns’ expressions

Low SVM decision scores at baseline predicted symptomatic remission at follow-up (FDR-corrected *P* value = 0.020). The same was true for functional recovery at follow-up (FDR-corrected *P* value = 0.034) and being (vs. not being) without antipsychotic medication (FDR-corrected *P* value = 0.019). Conversely, baseline BrainAGE does not predict any of these outcomes at follow-up. (see Fig. 4b and details in the Supplement).

### Univariate brain analyses

In the NFBC1966 sample, we found that the SVM decision score difference*timepoint related to the decreases in the hippocampus and medial prefrontal cortex, and increases that were located mainly in the periventricular white matter and pre- and post-central regions (Fig. 2). The analyses on other tissue types (details in the Supplementary Results) revealed that the SVM decision score difference * timepoint interaction reflected white matter atrophy in the regions that showed increased grey matter density (Supplementary Fig. 19). We found that the BrainAGE difference*timepoint interaction related to wide-spread decreases in the cortical and subcortical grey matter (Fig. 3c), and no positive interactions were found. The analyses on the global effects of the two multivariate patterns (Supplementary Fig. 12) revealed that the annual SVM decision score change over time did not relate to the annual change in global grey matter volume (*R*^2^ = 0.0006, *P* value = 0.81). However, the annual change in BrainAGE related to annual decreases in the global grey matter volume (*R*^2^ = 0.18, *P* value < 0.0001). This effect was even more pronounced in schizophrenia (R^2^ = 0.28, *P* value = 0.003).

### Supplementary results on the effect of disorder duration

We found an increment in the SVM decision scores over time in the NFBC1966 schizophrenia patients when we applied both the “short” (paired T-test, Cohen’s d = 0.47, t(28) = 2.53, *P* value = 0.017) and the “long” (Cohen’s d = 0.83, t(28) = 4.5, *P* value = 0.0001) disorder duration models to the NFBC1966. Similar findings were observed when we applied both the “short” (F(1, 88) = 11.1, *P* value = 0.001) and the “long” (F(1, 88) = 11.4, *P* value = 0.001) disorder duration models to the NFBC1966. Last, the CV-ratios of the “long” and “short” SZ-HC classifiers correlated across the brain (*ρ* = 0.55, *P* value < 0.0001).

## Discussion

We showed that a disorder-specific multivariate pattern expression magnifies in schizophrenia but not in controls leading to greater separation of patients with schizophrenia from controls as a function of time. This finding was not explained by longitudinal changes in non-disorder-specific brain aging, which possibly reflects co-occurring, albeit distinct, morphological brain processes in patients with schizophrenia. Our longitudinal setting in a prospective birth cohort demonstrated that our findings did not result from selection bias in recruiting patients with different disorder durations.

Our SZ-HC pattern trained on 144 COBRE individuals generalized to the NFBC1966 and two validation samples. This is in line with a previous study suggesting a minimum of 130 subjects to train a generalizable schizophrenia classifier^22^. The model did not generalize to MDD as these patients were mainly classified as controls, which implicates that the classifier is relatively disorder-specific. These findings are in line with previous studies reporting a separability between schizophrenia and depression^20^ and schizophrenia and bipolar disorder^21^. Future studies should extend our investigations on the cross-disorder generalizability of SZ-HC classifiers as many psychiatric and neurological disorders share similar brain network abnormalities^23^ and partly overlapping genetic underpinnings^24^. For example, future studies should explore whether the SZ-HC classifier generalizes to disorders with psychotic-like symptoms (e.g., a subgroup of frontotemporal dementia^25^).

The longitudinal magnification of SZ-HC pattern expression potentially reflects its progressive nature. The idea of schizophrenia as a progressive brain disorder has been controversially discussed since Kraepelin^26^ and is supported by widely-reported findings of chronic schizophrenia patients depicting more structural abnormalities than first-episode psychosis^27^. An alternative interpretation may be that our findings reflect the accumulation of secondary chronicity-related factors (e.g., cumulative antipsychotics exposure^5,28^), given the fact that the COBRE represented relatively chronic schizophrenia. Although our data cannot fully address this possibility, our supplementary analyses revealed that employing a model trained in a sample with a mean disorder duration of six years showed a similar divergence pattern of schizophrenia from controls over time compared to a training sample with a mean disorder duration of 27 years. Furthermore, we also found that this “short” disorder duration model used a brain pattern to classify schizophrenia from controls akin to the “long” disorder duration model. These findings might indicate that the schizophrenia brain pattern is qualitatively similar, at least to some extent, across schizophrenia patients of different ages, but its magnitude varies as a function of time.

The greater separation of schizophrenia from controls over time did not result from concomitant acceleration of brain aging in schizophrenia, although both patterns weighted some of the same brain regions. Specifically, although we replicated the previous finding of an association between schizophrenia patients’ expression of SZ-HC pattern and brain aging^13^ and acceleration of brain aging in schizophrenia over time compared to controls^17^, the change in brain aging between the baseline and the follow-up did not explain changes in SZ-HC pattern expression. Also, the progressive trajectory of schizophrenia patients’ disease fingerprint expression over time was present even after adjusting the statistical analysis for the effects of concurrent brain aging.

The increment in both patterns’ expression between the baseline and the follow-up reflected concomitant grey matter atrophy in the temporal and frontal lobe that are well-known structures to be affected over time in schizophrenia^6,29–31^. Despite these similarities, our results indicate that these patterns differ in terms of the magnitude of longitudinal grey matter atrophy. Specifically, we found that the annual increment in brain aging between the baseline and the follow-up explained up to 28% of the variance in annual grey matter volume reduction in schizophrenia, indicating that this pattern captures a large proportion of the global grey matter alterations. The longitudinal grey matter alterations leading to increment in SZ-HC pattern expression encompassed, conversely, only a few brain structures mainly located in the medial prefrontal cortex and hippocampus. Unlike brain aging, the expression increment in the SZ-HC pattern is also related to a progression in periventricular white matter atrophy that might encapsulate the well-reported progressive ventricular enlargement in schizophrenia over time^5,30,32^. Altogether, these findings suggest that the progressive brain changes in schizophrenia may relate in part to one multivariate pattern capturing circumscribed and relatively disorder-specific longitudinal brain changes, while another pattern captures global but non-disorder-specific longitudinal grey matter alterations.

Over time, the increment in schizophrenia detection over time was associated with the concurrent decline in verbal cognition and weight gain in schizophrenia. The SZ-HC pattern expression was also related to the number of hospitalizations and disorder duration, but further analyses revealed that concurrent brain aging effects explained these relationships. Surprisingly, the trajectory of psychotic symptoms or treatment-related factors had no significant association with SZ-HC pattern expression over the follow-up but were related to alterations in non-disorder-specific brain aging. Our findings of a negative association between SZ-HC pattern expression and poor cognitive performance are in line with the literature on wide-spread cognitive deficits in schizophrenia^33,34^ and the previous machine learning studies finding similar associations^35,36^. The positive relationship between BMI and SZ-HC pattern expression could reflect a high prevalence of obesity in schizophrenia^37^ but also disinhibition^38^, a common trait in schizophrenia^39^. Lastly, our data showed that misclassification of schizophrenia in baseline predicted symptomatic remission and functional recovery and being without any antipsychotic medication at the follow-up. Conversely, we found that brain aging at the baseline did not predict future disorder outcomes.

These findings might indicate that the schizophrenia brain pattern does not capture the course of primary schizophrenia symptoms and factors related to their treatment, as these variables were mainly linked to longitudinal changes in brain aging. The latter observation is keeping up with the accumulating evidence that brain aging captures the overall brain health, which in the present work could reflect exposure to antipsychotics and their effect on psychotic symptoms. Importantly, however, our results suggest that variations in SZ-HC pattern expression predict neurocognitive and outcome-related disease trajectories. The broad prognostic value of the SZ-HC pattern for these factors is in keeping with clinical evidence showing that cognitive performance is one of the strongest outcome predictors in schizophrenia^40–42^. Lastly, the divergence in the associations with the clinical variables between the two pattern expressions is in line with the clinical observation that cognitive deterioration is mostly separate from schizophrenia symptoms as alleviating these symptoms with antipsychotics does little for restoring cognitive deficits^42^.

Our study has several limitations. First, all machine learning models reflect their underlying sample. A training sample with different cumulative exposure to antipsychotics or cognitive profile might have resulted in slightly different findings. Second, our longitudinal NFBC1966 sample size was small. Future longitudinal large-scale studies are therefore needed to replicate our findings. The third limitation is that the analyses were conducted on established schizophrenia and, hence, do not apply to first-episode psychosis or clinical high-risk. Future longitudinal studies should extend our work by exploring the pattern trajectories in these groups. The fourth limitation is that an extensive neuropsychological battery was not available in the NFBC1966, limiting our conclusions regarding a broader multidimensional concept of neurocognition. Lastly, more timepoints in the longitudinal analysis would have enabled a more accurate characterization of the two multivariate patterns’ trajectories.

Despite these limitations, our research underlines the importance of taking account of longitudinal progression in disorder-specific pattern expression in schizophrenia when developing imaging-based machine learning classifiers to diagnose schizophrenia in different age groups. Consequently, care needs to be taken in future studies when aiming to develop one normative schizophrenia model for a sample with a wide age range.

## Methods

### Study samples

A detailed description is provided in the Supplement. The local ethical committees of each dataset have approved the data usage in the present study. The study participants of each dataset gave written informed consent. We chose the Centre for Biomedical Research Excellence (COBRE) of 70 schizophrenia and 74 controls as our model discovery dataset (Fig. 1a) due to its usage in previous schizophrenia-related neuroimaging studies and a wide age range^35,43,44^. Briefly, the dataset was collected by the Mind Research Network and the University of New Mexico. Schizophrenia diagnoses were defined using the Structured Clinical Interview for DSM-IV.

We used the Northern Finland Birth Cohort 1966 (NFBC1966)^45–47^ to explore the neuroanatomical separability of schizophrenia (*N* = 29) from controls (*N* = 61) as a function of time, using the COBRE-trained models. The NFBC1966 comprised the individuals born in 1966 in the two northernmost provinces in Finland^45^. Schizophrenia cases in the cohort were identified using nationwide registers. The controls were randomly selected individuals from the cohort without a psychotic disorder.

We used the Consortium for Neuropsychiatric Phenomics (CNP)^48^ and the Neuromorphometry by Computer Algorithm Chicago (NMorphCH; http://nunda.northwestern.edu/nunda/data/projects/NMorphCH)^49^ as validation samples to further assess the generalizability of the COBRE-trained models. The CNP was downloaded from the https://www.openfmri.org and the NMorphCH from the http://schizconnect.org. The CNP is a cross-sectional study, including individuals of different diagnoses: schizophrenia, bipolar disorder, and ADHD. In the present study, we used only schizophrenia (*N* = 18) and controls (*N* = 52). The NMorphCH (44 Schizophrenia, 43 controls) is a longitudinal study with a two-year follow-up.

To assess the cross-disorder performance of the schizophrenia classifier, we utilized two German Major depressive disorder (MDD) samples, one from Munich (103 MDD, 103 controls^13^) and the other from Münster^50,51^ (100 MDD, 100 controls).

We trained our brain aging model using the Information eXtraction from Images (IXI) (Fig. 1b), which includes 600 normal healthy individuals (https://brain-development.org/ixi-dataset/) from three sites in the U.K.

### Processing of the structural MRI

The structural MRI (sMRI) data were analyzed using VBM provided by the CAT12 toolbox version 1364 (Structural Brain Mapping Group, Jena University Hospital, Jena, Germany http://dbm.neuro.uni-jena.de/cat12/) on SPM12 in MATLAB r2017a. T1-weighted images were processed using the standard pipeline implemented in the toolbox, including denoising, skull stripping, spatial normalization to the Montreal Neurological Institute (MNI-152) space using the DARTEL algorithm, and segmentation into grey matter (GM), white matter, and cerebrospinal fluid. We conducted quality control by visually inspecting the processed images. Participants with MRI overall image quality worse than level C (i.e., satisfactory) from the CAT12 output were excluded from further analyses.

Due to differences in scanner types, we standardized each dataset using healthy control-based Z-normalization. In each dataset, each subject’s GM map was standardized using the Eq. (1):

1 $$z = \frac{{x_i - \mu _{HC}}}{{\sigma _{HC}}}$$

where x_i_ = individual subject’s GM map; $\mu _{HC}$ = average GM map based on controls; $\sigma _{HC}$ = standard deviation GM map based on controls.

### Schizophrenia vs. Controls (SZ-HC) classifier

We used the NeuroMiner (version 1.0, https://github.com/neurominer-git/NeuroMiner-1) running on MATLAB r2017a to train the SZ-HC classifier using support vector machines (SVMs) in a repeated, nested cross-validation framework consisting of an outer 10 × 10 -fold cross-validation cycle (CV2) and an inner 10 × 5-fold cross-validation cycle (CV1) (Fig. 1a). In the CV1, we used the following preprocessing steps: smoothing of the modulated GM maps with a full-width by half maximum (FWHM) smoothing kernel across a range of 4, 6, and 8 mm; correction for age and sex using a regression model computed in the healthy controls; principal component analysis for dimensionality reduction (80% variance retained); and scaling [0,1]. The training of the preprocessed features was conducted using a linear support vector machine (SVM; LIBSVM 3.1240; http://www.csie.ntu.edu.tw/~cjlin/libsvm). The winning models based on maximal balanced accuracy (i.e., the mean of sensitivity and specificity) across a range of SVM hyperparameters (0.0039, 0.0156, 0.0625, 0.25, 1, 4, 16, 64, and 256) in the CV1 cycle were applied to the respective out-of-training CV2 test subjects.

Next, without any in-between retraining, we applied the SZ-HC classifier to the NFBC1966, the replication data (the NMorphCH and the CNP), and the MDD data (MUC and Münster data). We determined a ROC-curve using SVM decision scores in the pooled out-of-training schizophrenia vs. controls samples and compared it to the MDD samples’ ROC-curves. The combined schizophrenia vs. controls sample consisted of the NFBC1966 (mean of SVM decision scores between the baseline and the follow-up), NMorphCH, and CNP. An Individual’s SVM decision score measures the distance from the decision boundary and can be viewed as denoting the degree of the classifier’s certainty of an individual’s class membership. We used SVM decision scores as a proxy of the magnitude of multivariate SZ-HC pattern expression.

### Brain aging regressor

We used support vector regression (SVR) in the IXI to estimate chronological age from sMRI. We used the same setup of repeated, nested cross-validation as in the above SZ-HC classification. In the CV1, we used the following preprocessing steps: smoothing of the modulated VBM images with an FWHM smoothing kernel of 8 mm; correction for sex and field strength (in Teslas) using a regression model; principal component analysis for dimensionality reduction (80% variance retained); and scaling [0,1]. Then, within each CV1 cycle, the preprocessed features were projected into a linear kernel space, where the SVR algorithm determined an optimal age-fitting function at a fixed C (regularization) parameter of 1 and a ν-parameter in the range of 0.2, 0.5, and 0.7. The winning models based on the lowest average Mean Absolute Error (MAE) in the CV1 cycle were applied to the respective out-of-training CV2 test subjects. In order to correct for the over- and underestimation of age in the lower and upper tails of the distribution, we calculated detrending parameters by fitting the CV2 subjects’ age residuals with their chronological age.

Next, we applied these brain aging models to the NFBC1966 baseline and follow-up data (Fig. 1b). Lastly, we calculated the brain age gap estimation (BrainAGE) score by subtracting chronological age from the estimated brain age. We used BrainAGE as a proxy of brain aging pattern expression.

### Clinical and outcome variables

A detailed description is provided in the Supplement. Cognition was assessed using the California Verbal Learning Test (CVLT)^52,53^, Abstraction, Inhibition and Working Memory task (AIM)^54^, and The Visual Object Learning Test (VOLT)^55^. Lifetime antipsychotic medication usage was converted to Chlorpromazine (CPZ) equivalents^56^. Positive and Negative Syndrome Scale (PANSS)^57^ was used to measure symptom dimensions. Social and Occupational Functioning Assessment Scale (SOFAS)^58^ and the clinical global impression (CGI) were assessed via interviews. The duration of the disorder and the number of hospitalizations were acquired from the medical records and nationwide healthcare registers.

We used the following disorder outcomes at the follow-up: being in remission, functional recovery (i.e., working or studying), being without antipsychotic medication. For symptomatic remission, we used the Andreasen criteria^59^ with modified duration criteria (no psychiatric hospital treatments six months before the follow-up).

### Statistical analyses

We used R version 3.6.1 (https://cran.r-project.org) accompanied with “pROC”^60^, “ggplot2”^61^, “lme4”^62^, “lmerTest”^63^, and “car”^64^ packages. Demographic characteristics were compared using the T-test, Mann-Whitney U-test, χ^2^-test, and Fisher’s exact test as specified in the Results section. Statistical outliers were defined as ± 3 SD. Missing data were imputed using the K-nearest neighbor imputation. The two models’ predictive performances were compared using DeLong’s test of the area under curve^65^ as specified in the Results section.

We used linear mixed models to investigate group by timepoint interaction on the SVM decision scores and BrainAGE. Also, we used linear mixed models to assess the relationships between the clinical variables with the two patterns’ expressions. The subject was modelled as random intercepts in the models. The coefficient of determination for the mixed effect model was calculated according to Jaeger et al. in the linear mixed models^66^. We used False Discovery Rate (FDR) Correction^67^ to control for false positives. Post hoc linear regression analyses on the clinical variable’s annual change on the change of a given pattern expression were performed in relationships where linear mixed-effects analyses showed a main effect.

We used a nonparametric permutation test to evaluate the statistical significance of the performance of a given multivariate pattern expression (i.e., SVM decision scores or BrainAGE) to predict future disorder outcomes. Specifically, we randomly resampled a given group label (e.g., remitted vs. non-remitted) and assessed the performance of predicting this resampled group label using the given pattern expression. Prediction performance was assessed with the area under curve (AUC). This procedure was performed 5,000 times, and the resulting values were used to make up the empirical null distribution. Finally, the observed AUC was compared against this null distribution using a two-sided test. FDR-correction was used to control false positives. The results are shown in Supplementary Figs. 13-14.

### Univariate brain analyses

For schizophrenia and brain aging patterns, we investigated the expression difference (i.e., between the timepoints) * timepoint interaction on the grey matter density using FSL version 6.0.1. Before the analysis, sex and TIV were regressed out from the GM using FSL’s GLM function. The regressed VBM maps were also spatially smoothed with 8 mm FWHM. In the FSL’s GLM, we modelled the interaction between SVM decision score differences and timepoint by using 2-way Mixed Effect ANOVA, where the within-subject effect was modelled as a random effect. We conducted a voxel-wise test to assess for statistical significance using the FSL’s^68^ randomise tool^69^ using 5000 iterations, applying threshold-free cluster enhancement (TFCE)^70^, and a family-wise error (FWE) correction to account for multiple voxel-wise comparisons. Statistical significant clusters were considered at *P* value < 0.05 (FWE-corrected).

Last, we investigated the effect of annual change in the expression of the SZ-HC and brain aging pattern expressions on the annual global grey matter volume reduction. Global grey matter volumes were acquired from the CAT12 processing output. The annual grey matter volume change was calculated using the difference in global grey matter volume between the timepoints divided by each individual’s exact length of follow-up. The association between the annual change in the expression of a given pattern and annual change in grey matter volume was assessed using linear regression.

### Data visualization

Multivariate GM patterns characterizing the decision boundaries (SZ vs. HC and brain aging) were visualized by back projecting each SVM/SVR model’s feature weight vector from PCA to MNI space, as described in Koutsouleris et al. (2015)^20^. The reliability of predictive voxels in the models was measured using a cross-validation ratio map with a threshold of ± 3. The CV-Ratio reflects the sum of the median weights across all CV1 folds divided by the standard error. Neuroimaging data were visualized using MRIcroGL (http://www.cabi.gatech.edu/mricrogl/). Statistical data were visualized using the “ggplot2”^61^ package in R version 3.6.1.

### Supplementary analyses

We investigated the possibility that the SZ-HC pattern trajectory in the NFBC1966 patients relates to the training sample’s disorder duration. This was done to partially address the possibility that our SZ-HC classifier mainly captures the disorder duration-related secondary factors, such as the well-replicated effects of cumulative antipsychotics on brain structures^5,28^. We trained, by using the same nested cross-validation design as in the primary analyses, one classifier with disorder duration below the median (“short,” i.e., below 13 years [Mean = 5.85, SD = 2.99], *N* = 68) and one with above the median (“long”, Mean = 26.82, SD = 9.66, *N* = 70) disorder duration in the COBRE. Controls were matched for sex and age for both schizophrenia subsamples. The two models were then applied to the NFBC1966.

### Reporting summary

Further information on research design is available in the Nature Research Reporting Summary linked to this article.

## Supplementary information

Supplementary Information

Reporting Summary

## Data Availability

The COBRE, the IXI, the CNP, and the NMoprhCH are publicly available datasets for research purposes. Investigators planning to use the NFBC 1966 data should apply through the procedures described at www.oulu.fi/nfbc. All relevant data are available from the authors upon a reasonable request.

## Code availability

The codes to reproduce the present study’s findings are available from the corresponding author upon a reasonable request.

## References

[1] Dwyer DB, Falkai P, Koutsouleris N (2018). Machine learning approaches for clinical psychology and psychiatry. Annu Rev. Clin. Psychol..

[2] Vieira S (2019). Using machine learning and structural neuroimaging to detect first episode psychosis: reconsidering the evidence. Schizophr Bull..

[3] Kambeitz J (2015). Detecting neuroimaging biomarkers for schizophrenia: a meta-analysis of multivariate pattern recognition studies. Neuropsychopharmacology.

[4] van Haren NEM (2007). Focal gray matter changes in schizophrenia across the course of the illness: a 5-year follow-up study. Neuropsychopharmacology.

[5] Fusar-Poli P (2013). Progressive brain changes in schizophrenia related to antipsychotic treatment? A meta-analysis of longitudinal MRI studies. Neurosci. Biobehav. Rev..

[6] Veijola J (2014). Longitudinal changes in total brain volume in schizophrenia: relation to symptom severity, cognition and antipsychotic medication. PLoS One.

[7] Jaaskelainen E (2013). A systematic review and meta-analysis of recovery in schizophrenia. Schizophr. Bull..

[8] Zipursky RB, Reilly TJ, Murray RM (2013). The myth of schizophrenia as a progressive brain disease. Schizophr. Bull..

[9] Davis KL (1998). Ventricular enlargement in poor-outcome schizophrenia. Biol. Psychiatry.

[10] Mitelman SA (2010). Progressive ventricular expansion in chronic poor-outcome schizophrenia. Cogn. Behav. Neurol..

[11] Franke K, Gaser C (2019). Ten years of BrainAGE as a neuroimaging biomarker of brain. Aging.: What Insights Have We Gained? Front. Neurol..

[12] Franke K, Luders E, May A, Wilke M, Gaser C (2012). Brain maturation: predicting individual BrainAGE in children and adolescents using structural MRI. Neuroimage.

[13] Koutsouleris N (2014). Accelerated brain aging in schizophrenia and beyond: a neuroanatomical marker of psychiatric disorders. Schizophr. Bull..

[14] Han LKM (2020). Brain aging in major depressive disorder: results from the ENIGMA major depressive disorder working group. Mol. Psychiatry.

[15] Elliott ML (2019). Brain-age in midlife is associated with accelerated biological aging and cognitive decline in a longitudinal birth cohort. Mol. Psychiatry.

[16] Cole JH (2018). Brain age predicts mortality. Mol. Psychiatry.

[17] Schnack HG (2016). Accelerated brain aging in Schizophrenia: a longitudinal pattern recognition study. Am. J. Psychiatry.

[18] Fjell AM (2015). Development and aging of cortical thickness correspond to genetic organization patterns. Proc. Natl Acad. Sci. USA.

[19] Lewis G, Pelosi AJ (1990). The case-control study in psychiatry. Br. J. Psychiatry.

[20] Koutsouleris N (2015). Individualized differential diagnosis of schizophrenia and mood disorders using neuroanatomical biomarkers. Brain.

[21] Schnack HG (2014). Can structural MRI aid in clinical classification? A machine learning study in two independent samples of patients with schizophrenia, bipolar disorder and healthy subjects. Neuroimage.

[22] Nieuwenhuis M (2012). Classification of schizophrenia patients and healthy controls from structural MRI scans in two large independent samples. Neuroimage.

[23] Crossley NA (2014). The hubs of the human connectome are generally implicated in the anatomy of brain disorders. Brain.

[24] Anttila, V. et al. Analysis of shared heritability in common disorders of the brain. Science 360, (2018). 10.1126/science.aap8757 PMC6097237 29930110

[25] Velakoulis D, Walterfang M, Mocellin R, Pantelis C, McLean C (2009). Frontotemporal dementia presenting as schizophrenia-like psychosis in young people: clinicopathological series and review of cases. Br. J. Psychiatry.

[26] DeLisi LE (2008). The concept of progressive brain change in schizophrenia: implications for understanding schizophrenia. Schizophr. Bull..

[27] Ellison-Wright I, Glahn DC, Laird AR, Thelen SM, Bullmore E (2008). The anatomy of first-episode and chronic schizophrenia: an anatomical likelihood estimation meta-analysis. Am. J. Psychiatry.

[28] Huhtaniska, S. et al. Long-term antipsychotic use and brain changes in schizophrenia—a systematic review and meta-analysis. *Hum. Psychopharmacol.* **32**, (2017). 10.1002/hup.2574 28370309

[29] Mathalon DH, Sullivan EV, Lim KO, Pfefferbaum A (2001). Progressive brain volume changes and the clinical course of schizophrenia in men: a longitudinal magnetic resonance imaging study. Arch. Gen. Psychiatry.

[30] van Haren NE (2008). Progressive brain volume loss in schizophrenia over the course of the illness: evidence of maturational abnormalities in early adulthood. Biol. Psychiatry.

[31] Guo JY (2015). Longitudinal regional brain volume loss in schizophrenia: relationship to antipsychotic medication and change in social function. Schizophr. Res.

[32] DeLisi LE, Sakuma M, Maurizio AM, Relja M, Hoff AL (2004). Cerebral ventricular change over the first 10 years after the onset of schizophrenia. Psychiatry Res.

[33] Mesholam-Gately RI, Giuliano AJ, Goff KP, Faraone SV, Seidman LJ (2009). Neurocognition in first-episode schizophrenia: a meta-analytic review. Neuropsychology.

[34] Fioravanti M, Carlone O, Vitale B, Cinti ME, Clare L (2005). A meta-analysis of cognitive deficits in adults with a diagnosis of schizophrenia. Neuropsychol. Rev..

[35] Cabral C (2016). Classifying Schizophrenia using multimodal multivariate pattern recognition analysis: evaluating the impact of individual clinical profiles on the neurodiagnostic performance. Schizophrenia Bull..

[36] de Pierrefeu A (2018). Identifying a neuroanatomical signature of schizophrenia, reproducible across sites and stages, using machine learning with structured sparsity. Acta Psychiatr. Scand..

[37] Vancampfort D (2013). A meta-analysis of cardio-metabolic abnormalities in drug naïve, first-episode and multi-episode patients with schizophrenia versus general population controls. World Psychiatry.

[38] Amlung M, Petker T, Jackson J, Balodis I, MacKillop J (2016). Steep discounting of delayed monetary and food rewards in obesity: a meta-analysis. Psychological Med..

[39] Ouzir M (2013). Impulsivity in schizophrenia: a comprehensive update. Aggression Violent Behav..

[40] Hofer A (2011). Symptomatic remission and neurocognitive functioning in patients with schizophrenia. Psychol. Med.

[41] Sánchez P (2009). Predictors of longitudinal changes in schizophrenia: the role of processing speed. J. Clin. Psychiatry.

[42] Green MF, Harvey PD (2014). Cognition in schizophrenia: past, present, and future. Schizophr. Res Cogn..

[43] Morgan SE (2019). Cortical patterning of abnormal morphometric similarity in psychosis is associated with brain expression of schizophrenia-related genes. Proc. Natl Acad. Sci. USA.

[44] Monté-Rubio GC, Falcón C, Pomarol-Clotet E, Ashburner J (2018). A comparison of various MRI feature types for characterizing whole brain anatomical differences using linear pattern recognition methods. Neuroimage.

[45] Rantakallio P (1969). Groups at risk in low birth weight infants and perinatal mortality. Acta Paediatr. Scand..

[46] Isohanni M (1997). A comparison of clinical and research DSM-III-R diagnoses of schizophrenia in a Finnish national birth cohort. Clinical and research diagnoses of schizophrenia. Soc. Psychiatry Psychiatr. Epidemiol..

[47] Jääskeläinen, E. et al. Twenty years of Schizophrenia research in the Northern Finland birth cohort 1966: a systematic review. *Schizophr Res. Treat.* 2015, 524875 (2015). 10.1155/2015/524875 PMC4452001 26090224

[48] Poldrack RA (2016). A phenome-wide examination of neural and cognitive function. Sci. Data.

[49] Wang L (2016). SchizConnect: Mediating neuroimaging databases on schizophrenia and related disorders for large-scale integration. Neuroimage.

[50] Kircher, T. et al. Neurobiology of the major psychoses: a translational perspective on brain structure and function-the FOR2107 consortium. *Eur. Arch. Psychiatry Clin. Neurosci.*10.1007/s00406-018-0943-x (2018). 10.1007/s00406-018-0943-x 30267149

[51] Vogelbacher C (2018). The Marburg-Munster Affective Disorders Cohort Study (MACS): A quality assurance protocol for MR neuroimaging data. Neuroimage.

[52] Delis, D., Kramer, J., Ober, B. & Kaplan, E. The California verbal learning test: administration and interpretation. San Antonio, TX: Psychological Corporation (1987).

[53] Stone WS (2011). Group and site differences on the California Verbal Learning Test in persons with schizophrenia and their first-degree relatives: findings from the Consortium on the Genetics of Schizophrenia (COGS). Schizophr. Res.

[54] Glahn DC, Cannon TD, Gur RE, Ragland JD, Gur RC (2000). Working memory constrains abstraction in schizophrenia. Biol. Psychiatry.

[55] Glahn DC, Gur RC, Ragland JD, Censits DM, Gur RE (1997). Reliability, performance characteristics, construct validity, and an initial clinical application of a visual object learning test (VOLT). Neuropsychology.

[56] Andreasen NC, Pressler M, Nopoulos P, Miller D, Ho B-C (2010). Antipsychotic dose equivalents and dose-years: a standardized method for comparing exposure to different drugs. Biol. Psychiatry.

[57] van der Gaag M (2006). The five-factor model of the Positive and Negative Syndrome Scale I: confirmatory factor analysis fails to confirm 25 published five-factor solutions. Schizophr. Res.

[58] Rybarczyk, B. Social and Occupational Functioning Assessment Scale (SOFAS). in Encyclopedia of Clinical Neuropsychology (eds. Kreutzer, J. S., DeLuca, J. & Caplan, B.) 2313–2313 (Springer New York, 2011). 10.1007/978-0-387-79948-3_428.

[59] Andreasen NC (2005). Remission in schizophrenia: proposed criteria and rationale for consensus. Am. J. Psychiatry.

[60] Robin X (2011). pROC: an open-source package for R and S+ to analyze and compare ROC curves. BMC Bioinforma..

[61] Wickham, H. ggplot2: elegant graphics for data analysis. Springer New York. (2009).

[62] Bates D, Mächler M, Bolker B, Walker S (2015). Fitting linear mixed-effects models using lme4. J. Stat. Softw..

[63] Kuznetsova, A., Brockhoff, P., Christensen, R. lmerTest package: tests in linear mixed effects models. *J. Stat. Softw*. **82**, (2017).

[64] Fox, J. & Weisberg, S. An {R} Companion to applied regression, Second Edition. Thousand Oaks CA: Sage. http://socserv.socsci.mcmaster.ca/jfox/Books/Companion. (2011).

[65] DeLong ER, DeLong DM, Clarke-Pearson DL (1988). Comparing the areas under two or more correlated receiver operating characteristic curves: a nonparametric approach. Biometrics.

[66] Jaeger BC, Edwards LJ, Das K, Sen PK (2017). An R2 statistic for fixed effects in the generalized linear mixed model. J. Appl. Stat..

[67] Benjamini Y, Hochberg Y (1995). Controlling the false discovery rate: a practical and powerful approach to multiple testing.. J. R. Stat. Soc. Ser. B (Methodol.).

[68] Jenkinson M, Beckmann CF, Behrens TE, Woolrich MW, Smith SM (2012). Fsl. Neuroimage.

[69] Nichols TE, Holmes AP (2002). Nonparametric permutation tests for functional neuroimaging: a primer with examples. Hum. Brain Mapp..

[70] Smith SM, Nichols TE (2009). Threshold-free cluster enhancement: addressing problems of smoothing, threshold dependence and localisation in cluster inference. Neuroimage.

