# Supplementary material for: The progression of disorder-specific brain pattern expression in schizophrenia over 9 years
Source: NPJ Schizophr. 2021 Jun 14;7:32. doi: 10.1038/s41537-021-00157-0 (PMC8203625; doi:10.1038/s41537-021-00157-0)
Supplement: Supplementary file 1 — Supplementary Information [file 41537_2021_157_MOESM1_ESM.pdf]

The progression of disorder-specific brain pattern expression in schizophrenia over nine years

Supplementary material:

Supplementary Methods
Supplementary Results
Supplementary Tables 1-4
Supplementary Figures 1-19

Johannes Lieslehto, MD, PhD[1,7,11,*]; Erika Jääskeläinen, MD, PhD[1,2,5]; Vesa Kiviniemi, MD, PhD[4,5]; Marianne Haapea, PhD [1,2,5]; Peter B. Jones, MD, PhD [6]; Graham K. Murray MD PhD [6]; Juha Veijola MD PhD [2,5]; Udo Dannlowski MD, PhD[8]; Dominik Grotegerd PhD[8]; Susanne Meinert PhD[8]; Tim Hahn PhD[8]; Anne Ruef, PhD[7]; Matti Isohanni, MD, PhD[1]; Peter Falkai, MD, PhD[7]; Jouko Miettunen, PhD[1,5]; Dominic Dwyer, PhD[7,C]; Nikolaos Koutsouleris, MD, PhD[7,9,10,C]

[1] Center for Life Course Health Research, University of Oulu, Oulu, Finland
[2] Department of Psychiatry, Oulu University Hospital, Oulu, Finland
[3] Department of Psychiatry, Research Unit of Clinical Neuroscience, University of Oulu, Oulu, Finland
[4] Department of Diagnostic Radiology, Oulu University Hospital, Oulu, Finland
[5] Medical Research Center Oulu, Oulu University Hospital and University of Oulu, Oulu, Finland
[6] Department of Psychiatry, University of Cambridge, Cambridge, UK
[7] Department of Psychiatry and Psychotherapy, Ludwig-Maximilian-University, Munich, Germany
[8] Department of Psychiatry, University of Münster, Germany
[9] International Max Planck Research School for Translational Psychiatry, Germany
[10] Institute of Psychiatry, Psychology and Neuroscience, King's College London, UK
[11] Department of Forensic Psychiatry, University of Eastern Finland, Niuvanniemi Hospital, Kuopio, Finland

[C] These authors contributed equally

[*]Correspondence: Dr. Johannes Lieslehto; Center for Life Course Health Research, University of Oulu, P.O. Box 5000, 90014 Oulu, Finland; e-mail: johannes.lieslehto@niuva.fi, +358 29 5242227

Supplementary Methods

Description of the COBRE

The dataset includes 71 schizophrenia and 74 controls. The exclusion criteria of the dataset were: a history of neurological disorder or mental retardation, past severe head trauma with more than 5 minutes loss of consciousness, history of substance abuse or dependence within the last 12 months. Schizophrenia diagnoses were evaluated using the Structured Clinical Interview for DSM-IV. The dataset was downloaded from the COINS database (https://coins.trendscenter.org).

Structural MRI was conducted using 3T SIEMENS MAGNETOM TrioTim syngo MR B17, and a multi-echo MPRAGE (MEMPR) sequence with the following parameters: TR = 2530, TE = (1.64, 3.5, 5.36, 7.22, 9.08), TI = 900 ms, flip angle = 7°, FOV = 256x256 mm, slab thickness = 176 mm, matrix = 256x256x176, voxel size = 1 x 1 x 1 mm, number of echos = 5, pixel bandwidth = 650 Hz, Total scan time = 6 min. As part of the MRI quality control (described in the *Processing of the T1-weighted images* section*)*, we excluded one schizophrenia patient, leaving 70 schizophrenia and 74 controls.

Description of the NFBC1966

The NFBC1966 is an unselected population birth cohort based on 12 058 deliveries in the two Northernmost provinces in Finland (Oulu and Lapland). We used the nationwide Finnish Hospital Discharge Register (FHDR, currently known as the Care Register for Health Care, https://thl.fi/en/web/thlfi-en/statistics/information-on-statistics/register-descriptions/care-register-for-health-care) to identify the NFBC1966 members with a psychotic disorder. This register has data on patients discharged from hospitals since 1969. Thus, we did not draw schizophrenia cases from psychiatric services. In the baseline, we validated all schizophrenia diagnoses according to the Diagnostic and Statistical Manual of Mental Disorders Third Edition Revised (DSM-III-R) criteria. In the follow-up, we used SCID-IV as a diagnostic instrument, supplemented by anamnestic information (including hospital medical records until the year 2009). Besides, we selected random control participants from the same birth cohort. The only inclusion criterion was that these participants had no history of a psychotic episode. The flow-chart presents the selection of the participants of the NFBC1966 in the present study (Supplementary Figure 1). All the study participants gave written informed consent. We excluded individuals with severe head trauma

(based on register data and interviews), psychotic syndromes other than schizophrenia, a neurological disorder with a potential effect on brain structure, and poor-quality sMRI at baseline or the follow-up.

We used the same 1.5 T GE Signa (General Electric, Milwaukee, Wisconsin) at both the baseline (34y) and the follow-up (43y). Note, however, that there was an update in the scanner electronics and the sequence between the timepoints, as described below. At the baseline, we obtained T1-weighted high-resolution three-dimensional spoiled gradient echo (3D SPGR) using the following parameters: coronal plane covering the whole brain (slice thickness 1.5 mm), in-plane resolution matrix size 256 x 256, voxel size 1.5 mm x 1 mm x 1 mm; TR 35 ms; TE 5 ms, flip angle = 35. Between the timepoints, the scanner was upgraded into HDxt with a new gradient system and parallel image data acquisition with an eight-channel receiving coil. At the follow-up, we obtained the T1 weighted images using a 3D fast spoiled gradient echo (FSPGR) sequence with the following parameters: slice thickness = 1 mm; in-plane resolution matrix size 256 x 256; voxel size 1 mm x 1 mm x 1 mm, TR = 12.576 ms, TE = 5.3 ms and flip angle =20.

Schizophrenia and control participants completed the California Verbal Learning Test (CVLT)[1], Abstraction, Inhibition and Working Memory task (AIM)[2], and The Visual Object Learning Test (VOLT)[3] at both timepoints. In the CVLT, we used the total score of the Immediate free recall of trials 1–5 since this score has been demonstrated as having the most significant effect size of the CVLT variables in detecting verbal learning deficits in schizophrenia [4]. AIM results in two outcome measures: total score of the abstraction trials (AIM-) and total score of trials with abstraction and memory (AIM+). VOLT measures visual-spatial learning and memory analogous.

A detailed description of the lifetime antipsychotic medication calculation is described in Moilanen et al. (2016)[5]. Briefly, lifetime antipsychotic medication usage was collected during 2008–2012 using all available hospital and outpatient records, extended by the interviews during the follow-up and the nationwide register data. For comparability, we converted antipsychotic medication doses into chlorpromazine equivalents (CPZ)[6].

Positive and Negative Syndrome Scale (PANSS)[7] was used to measure symptom dimensions from one week before the baseline and the follow-up. At the baseline, PANSS was acquired from the SCID

I diagnostic interview. At the follow-up, PANSS was acquired from a PANSS specific interview. SOFAS and Clinical Global Impression scale (CGI) were assessed via interviews. The duration of the disorder was acquired from the medical records and registers. The number of hospitalizations was acquired from the nationwide registers and was used as a proxy of relapse.

Description of the validation datasets

We used two open datasets, namely the Consortium for Neuropsychiatric Phenomics (CNP)[8] and the Neuromorphometry by Computer Algorithm Chicago (NMorphCH)[9]. The CNP was downloaded from the OpenfMRI (https://www.openfmri.org) and the NMorphCH from the Schizconnect (http://schizconnect.org).

The NMorphCH (44 Schizophrenia and 43 controls) is a longitudinal study examining the clinical, cognitive, and neuroimaging (MRI) data from schizophrenia and control subjects at baseline and after two years. Schizophrenia diagnoses were acquired using DSM-IV. The mean age in schizophrenia was 32.5 (SD = 6.9) and 31.5 (SD = 8.4) in controls. 29 schizophrenia and 24 controls in the NMorphCH had available longitudinal data. In the NMorphCH, sMRI was conducted with 3 T using the following parameters: TR = 3.15 ms, TE = 1.37 ms, flip angle = 8°, 160 x 160 matrix, 128 slices, slice thickness = 1.6 mm.

The CNP participants were ages 21-50 and were recruited by community advertisements from the Los Angeles area. Schizophrenia diagnosis was verified with the SCID-IV. Due to different scanner types, we utilized only those subjects in the CNP that were imaged using Siemens version Syngo MR B15, leaving 52 controls without any disorder (mean age = 30.7, SD = 9.13) and 18 schizophrenia patients (mean age = 36.8, SD = 8.7). The MPRAGE in the CNP were imaged with 3 T using the following parameters: TR = 1.9 s, TE = 2.26 ms, FOV = 250 mm, matrix =256 × 256, sagittal plane, slice thickness = 1 mm, 176 slices.

Description of the MDD data

In the Munich sample (MUC) [10], patients with major depression (N = 103) were examined at the Department of Psychiatry and Psychotherapy, Ludwig-Maximilian-University Munich (LMU) using the Structured Clinical Interview for DSM-IV and the Hamilton Depression Rating Scale. The exclusion criteria were insufficient knowledge of German or a history of neurological disorders (e.g.,

dementia), somatic disorders affecting the central nervous system, personality disorders, substance abuse or dependence, anorexia nervosa, or mental disability (IQ<70). The mean age in the MDD group was 42.1, SD = 11.9. We also utilized 103 sex and age-matched control participants. The control participants' exclusion criteria included a history of head trauma, cortisol treatments, somatic conditions affecting the central nervous system, present or past alcohol abuse, and a personal or familial history of psychiatric disorders in first-degree relatives. Structural MRI scanning was conducted using the Siemens MAGNETOM Vision 1.5T. The 3D-MPRAGE sequence was conducted using the following parameters: TE, 4.9ms; TR, 11.6ms; the field of view, 230mm; matrix, 512×512×126 contiguous axial slices; voxel dimensions, 0.45×0.45×1.5mm.

We also utilized the Münster site in the Marburg-Münster Affective Disorders Cohort Study (referred to hereinafter as Münster dataset) [11,12]. For the present study, we utilized 100 MDD and 100 sex and age-matched control participants (mean age = 30.5, SD = 8.0). MDD diagnosis was acquired with the SCID interview according to the DSM-IV criteria. The study's exclusion criteria were verbal IQ < 80, substance-related disorders, history of severe neurological or medical disorders, and current benzodiazepine use. Structural MRI data were acquired at a 3T MRI scanner (Prisma, Siemens, Erlangen, Germany) with a 20-channel head matrix Rx-coil using MPRAGE with the following parameters: slice thickness = 3.8 mm; effective voxel size 3.28 mm x 3.28 mm x 3.28 mm, TR = 2000 ms, TE = 29 ms and flip angle = 90.

Description of the IXI dataset

This sample includes 600 form normal healthy individuals (downloaded from https://brain-development.org/ixi-dataset/). The sMRI was conducted at three sites in the UK: Hammersmith Hospital using a Philips 3T system (TR = 9.60000038146972, TE = 4.60269975662231, Number of Phase Encoding Steps = 208, Echo Train Length = 208, Reconstruction Diameter = 240.0, Acquisition Matrix = 208 x 208, Flip Angle = 8.0), Guy's Hospital using a Philips 1.5T system (TR = 9.813, TE = 4.603, Number of Phase Encoding Steps = 192, Echo Train Length = 0, Reconstruction Diameter = 240, Flip Angle = 8) and Institute of Psychiatry using a GE 1.5T system (scanning parameters not available). After the quality control (QC), we retained 561 subjects (with ages ranging from 20 to 86 years) for the SVR.

Supplementary Results

The effect of attrition rate in the NFBC1966 on the SVM decision scores

Although the non-participating schizophrenia patients had higher SVM decision scores (mean=0.91; SD=2.63) vs. participating schizophrenia patients (mean=0.41; SD=1.53), there were no statistically significant differences (Cohen's d=0.25, *t*(21)=0.70, P-value=0.49). Further, the participating (vs. non-participating) subjects did not differ in positive (Cohen's d=0.043, *t*(24)=-0.13, P-value=0.90), negative (Cohen's d=0.3, *t*(25)=-0.89, P-value=0.38) or general symptoms (Cohen's d=0.046, *t*(19)=0.12, P-value=0.90). However, the non-participating patients had been exposed to lower amount of CPZ dose years compared to participating schizophrenia patients (Cohen's d=0.63, *t*(36)=-2.57, P-value=0.014).

The effect of image quality on the prediction performance

There were no differences (T-tests, all P-values>0.1) between those who were correctly (vs. not correctly) classified as schizophrenia or controls in comparison to the corresponding image quality. Even when we combined the COBRE and the NFBC1966 baseline, there were no differences in classification error with respect to image quality (Cohen's d=0.16, *t*(106)=-1.09, P-value=0.28). The same was true when the COBRE sample was combined with the NFBC1966 follow-up (Cohen's d=0.16, *t*(95)=-1.03, P-value=0.31). These comparisons are shown in Supplementary Figure 3.

The longitudinal changes in SVM decision scores in the NMorphCH validation sample

Using those participants of the NMorphCH with 2-year follow-up data (29 schizophrenia and 24 controls), we found that schizophrenia patients' SVM decision scores changed into more schizophrenia-likeness, but this change was not significant (Cohen's d=0.11, t(28)=0.61, P-value=0.55). Furthermore, we found no timepoint by group interaction on the SVM decision scores (*F*(1,51)=0.32, P-value=0.57). This is shown in Supplementary Figure 16.

Classification of schizophrenia vs. controls using the NFBC1966

We used the same nested cross-validation design at the baseline and the follow-up as in the main analyses in the COBRE, but now all the analyses were conducted in the NFBC1966. At the baseline, our classification of schizophrenia from controls resulted in a BAC of 66.7% (sensitivity=44.7%, specificity=77.0%). This model's prediction performance (AUC=0.678) did not differ from the

performance predicted using the COBRE-trained models with OOCV at the baseline (AUC=0.76), DeLong's test for two correlated ROC curves: Z = 1.0639, P-value = 0.287. At the follow-up, our classification of schizophrenia from controls resulted in a BAC of 78.0% (sensitivity=72.4%, specificity=83.6%). The prediction performance (AUC= 0.87) did not differ from the results predicted using the COBRE-models at the follow-up (AUC=0.87), DeLong's test for two correlated ROC curves: Z = -0.087, p-value = 0.93. The corresponding ROC-curves for the schizophrenia vs. controls classification using the NFBC1966 are provided in Supplementary Figure 17.

"Long" vs. "short" disorder duration models of the COBRE

The mean age of the "short" disorder duration subsample of the COBRE (i.e., disorder duration below the median) schizophrenia was 26.7 (SD=8.7) and in the "long" disorder duration subsample of the COBRE (i.e., disorder duration above the median) 48.3 (8.7), T-test t(66.9)=10.26, Cohen’s d=2.47, P-value<0.0001. Using the "long" disorder duration models, our classification of schizophrenia from controls resulted in a BAC of 64.3% (sensitivity=60%, specificity=68.6%). Using the "short" disorder duration models, our classification of schizophrenia from controls resulted in a BAC of 54.4% (sensitivity=41.2%, specificity=67.6%). The ROC curves are provided in Supplementary Figure 18. There was a trend towards greater AUC in the "long" disorder duration model (AUC=0.7) compared to the "short" disorder duration (AUC=0.57), DeLong's test D = -1.3705, one-tailed P-value = 0.086. Further, SVM decision scores were higher in the schizophrenia patients of the COBRE that were used to train the "long" disorder duration model vs. schizophrenia that were used to train the "short" disorder duration model (Cohen’s d=0.50, *t*(45)=2.08, one-tailed P-value=0.043).

The effect of disorder duration of the COBRE sample on the prediction performance in the NFBC1966

We applied the "long" and "short" disorder duration models to the NFBC1966 using OOCV. Both of these disorder duration models were based on half the COBRE sample and, therefore, were less than the recommended 130 subjects required to train a generalizable model [21]. Thus, these explorative analyses were considered proof of concept to investigate whether the divergence in a change in SVM decision scores between schizophrenia and controls is observed regardless of the training sample's disorder duration.

Employing the "short" disorder duration models, we found better performance at the follow-up (Sensitivity=51.7%, Specificity=96.7%, BAC=74.2%, AUC=0.83) compared to the baseline

(Sensitivity=37.9%, Specificity= 95.1%, BAC=66.5%, AUC=0.72) using paired DeLong's test of AUC-curves (Z=2.19, P-value=0.029). The SVM decision scores varied increased over time in the NFBC1966 schizophrenia patients (paired T-test, Cohen's d=0.47, $t(28)=2.53$, P-value=0.017). We also found an SVM decision score by timepoint interaction ($F(1,88)=11.1$, P-value=0.001).

Employing the "long" models, we found better performance at the follow-up (Sensitivity=41.4%, Specificity=98.4%, BAC=69.9%, AUC=0.85) compared to the baseline (Sensitivity=20.7%, Specificity= 100%, BAC=60.3%, AUC=0.70) using paired DeLong's test (Z=2.82, P-value=0.005). Again, the SVM decision scores increased over time in the NFBC1966 schizophrenia patients (Cohen's d=0.83, $t(28)=4.5$, P-value=0.0001). We also found an SVM decision score by timepoint interaction ($F(1,88)=11.4$, P-value=0.001).

Using DeLong's test of paired AUC-curves, there was no difference in performance when comparing "long" vs. "short" (Z = -0.24, P-value=0.81) at the NFBC1966 follow-up. At the baseline, there was also no difference in performance when comparing "long" vs. "short" models (Z=0.534, P-value=0.59). The ROC-curves are provided in Supplementary Figure 19.

*SVM decision score difference*timepoint interaction on the white matter density*

Due to the relatively unexpected finding that SVM decision score difference*timepoint interaction related to the increases in the periventricular white matter of the grey matter maps, we explored whether this finding stems from partial volume effect. We suspected that we might detect a false increase in grey matter signal in the white matter if the grey matter boundary moves into white matter due to white matter atrophy over time. This was confirmed as we found that SVM decision score difference*timepoint related to decreases of white matter in the same regions that showed increases in the grey matter density contrast (Supplementary Figure 12).

The relationship between BMI and CVLT with the SVM decision scores in the controls of the NFBC1966

Given that both CVLT and BMI are related to SVM decision scores in schizophrenia, we also tested whether similar relationships is found for the control subjects of the NFBC1966. Across the timepoints, we found no relationship between CVLT and SVM decision scores (F(1,104)=0.51, P-

value=0.48) and no relationship between BMI and SVM decision scores ($F(1,89)=0.73$, P-value=0.40) in controls.

Supplementary References:

1. Delis D, Kramer J, Ober B, Kaplan E. The California verbal learning test: administration and interpretation. *San Antonio, TX: Psychological Corporation*. Published online 1987.

2. Glahn DC, Cannon TD, Gur RE, Ragland JD, Gur RC. Working memory constrains abstraction in schizophrenia. *Biological Psychiatry*. 2000;47(1):34-42. doi:10.1016/S0006-3223(99)00187-0

3. Glahn DC, Gur RC, Ragland JD, Censits DM, Gur RE. Reliability, performance characteristics, construct validity, and an initial clinical application of a visual object learning test (VOLT). *Neuropsychology*. 1997;11(4):602.

4. Stone WS, Giuliano AJ, Tsuang MT, et al. Group and site differences on the California Verbal Learning Test in persons with schizophrenia and their first-degree relatives: findings from the Consortium on the Genetics of Schizophrenia (COGS). *Schizophr Res*. 2011;128(1-3):102-110. doi:10.1016/j.schres.2011.01.005

5. Moilanen JM, Haapea M, Jääskeläinen E, et al. Long-term antipsychotic use and its association with outcomes in schizophrenia – the Northern Finland Birth Cohort 1966. *European Psychiatry*. 2016;36:7-14. doi:10.1016/j.eurpsy.2016.03.002

6. Andreasen NC, Pressler M, Nopoulos P, Miller D, Ho B-C. Antipsychotic dose equivalents and dose-years: a standardized method for comparing exposure to different drugs. *Biol Psychiatry*. 2010;67(3):255-262. doi:10.1016/j.biopsych.2009.08.040

7. van der Gaag M, Cuijpers A, Hoffman T, et al. The five-factor model of the Positive and Negative Syndrome Scale I: confirmatory factor analysis fails to confirm 25 published five-factor solutions. *Schizophr Res*. 2006;85(1-3):273-279. doi:10.1016/j.schres.2006.04.001

8. Poldrack RA, Congdon E, Triplett W, et al. A phenome-wide examination of neural and cognitive function. *Scientific Data*. 2016;3(1):160110. doi:10.1038/sdata.2016.110

9. Wang L, Alpert KI, Calhoun VD, et al. SchizConnect: Mediating neuroimaging databases on schizophrenia and related disorders for large-scale integration. *Neuroimage*. 2016;124(Pt B):1155-1167. doi:10.1016/j.neuroimage.2015.06.065

10. Koutsouleris N, Davatzikos C, Borgwardt S, et al. Accelerated brain aging in schizophrenia and beyond: a neuroanatomical marker of psychiatric disorders. *Schizophr Bull*. 2014;40(5):1140-1153. doi:10.1093/schbul/sbt142

11. Kircher T, Wohr M, Nenadic I, et al. Neurobiology of the major psychoses: a translational perspective on brain structure and function-the FOR2107 consortium. *Eur Arch Psychiatry Clin Neurosci*. Published online September 28, 2018. doi:10.1007/s00406-018-0943-x

12. Vogelbacher C, Mobius TWD, Sommer J, et al. The Marburg-Munster Affective Disorders Cohort Study (MACS): A quality assurance protocol for MR neuroimaging data. *Neuroimage*. 2018;172:450-460. doi:10.1016/j.neuroimage.2018.01.079

13. Jenkinson M, Beckmann CF, Behrens TE, Woolrich MW, Smith SM. Fsl. *Neuroimage*. 2012;62(2):782-790. doi:10.1016/j.neuroimage.2011.09.015

14. Nichols TE, Holmes AP. Nonparametric permutation tests for functional neuroimaging: a primer with examples. *Hum Brain Mapp*. 2002;15(1):1-25. doi:10.1002/hbm.1058

15. Smith SM, Nichols TE. Threshold-free cluster enhancement: addressing problems of smoothing, threshold dependence and localisation in cluster inference. *Neuroimage*. 2009;44(1):83-98. doi:10.1016/j.neuroimage.2008.03.061

16. Koutsouleris N, Meisenzahl EM, Borgwardt S, et al. Individualized differential diagnosis of schizophrenia and mood disorders using neuroanatomical biomarkers. *Brain*. 2015;138(Pt 7):2059-2073. doi:10.1093/brain/awv111

17. Wickham H. ggplot2: elegant graphics for data analysis. Springer New York. 2009;(Journal Article).

18. DeLong ER, DeLong DM, Clarke-Pearson DL. Comparing the areas under two or more correlated receiver operating characteristic curves: a nonparametric approach. *Biometrics*. 1988;44(3):837-845.

19. Jaeger BC, Edwards LJ, Das K, Sen PK. An R2 statistic for fixed effects in the generalized linear mixed model. *Journal of Applied Statistics*. 2017;44(6):1086-1105. doi:10.1080/02664763.2016.1193725

20. Benjamini Y, Hochberg Y. Controlling the False Discovery Rate: A Practical and Powerful Approach to Multiple Testing. *Journal of the Royal Statistical Society Series B (Methodological)*. 1995;57(1):289-300.

21. Nieuwenhuis M, van Haren NEM, Hulshoff Pol HE, Cahn W, Kahn RS, Schnack HG. Classification of schizophrenia patients and healthy controls from structural MRI scans in two large independent samples. *Neuroimage*. 2012;61(3):606-612. doi:10.1016/j.neuroimage.2012.03.079

Supplementary Table 1. Performance of the COBRE trained SVM models in the NFBC1966, replication samples, and the MDD samples

Sample	Disorder	Sensitivity %	Specificity %	AUC	BAC %	TP	TN	FP	FN
NFBC1966 (*BL*)	SZ-HC	58.6	86.9	0.76	72.8	17	53	8	12
NFBC1966 (*FU*)	SZ-HC	75.9	83.6	0.87	79.7	22	51	10	7
NMorphCH	SZ-HC	54.5	83.7	0.82	69.1	24	36	7	20
CNP	SZ-HC	72.2	82.7	0.81	77.5	13	43	9	5
MUC	MDD-HC	18.4	79.6	0.52	49	19	82	21	84
Münster	MDD-HC	35.0	88.0	0.63	61.5	35	88	12	65

Abbreviations: BL, Baseline; FU, Follow-up; MUC, Munich; AUC, Area Under Curve; BAC, Balanced Accuracy; TP, True Positive; TN, True Negative; FP, False Positive; FN, False Negative; SZ-HC, Schizophrenia vs. Controls classification; MDD-HC, Depression vs. Controls classification

Supplementary Table 2. SVM decision score difference*timepoint interaction on the grey matter density. FSL's Randomise, TFCE (Threshold-Free Cluster Enhancement)-corrected p<0.05. Coordinates are in MNI-space.

Voxels	T-stat	X (MNI)	Y (MNI)	Z (MNI)	Anatomical structure
10173	8.17	-36	-24	63	Left Postcentral Cortex/White matter
276	4.32	21	-96	-21	Occipital pole
30	3.53	15	-48	24	Left cerebral white matter
232	-5.07	27	-9	-39	Right Parahippocampal gyrus
169	-5.04	-6	36	-18	Inferior medial Frontal Cortex
142	-4.56	-33	-27	-18	Left Hippocampus
79	-4.17	3	42	21	Paracingulate Cortex
57	-4.57	42	42	0	Right Frontal Pole
24	-4.37	54	6	9	Right Inferior Frontal Gyrus
19	-4.08	21	54	-9	Right Frontal Pole

Supplementary Table 3. Linear mixed-effect models to explore the associations between SVM decision scores and clinical variables

Variable	R^2	Estimate	Std. Error	df	T-value	P-value	P-value (FDR)
CVLT	0.204	-0.040	0.013	39.437	-3.103	0.004	0.012
VOLT	<0.001	-0.004	0.026	48.757	-0.146	0.884	0.929
AIM+	<0.001	0.005	0.053	37.892	0.089	0.929	0.929
AIM-	0.001	0.012	0.041	34.448	0.283	0.779	0.909
SOFAS	0.010	-0.009	0.013	54.849	-0.715	0.478	0.743
CPZ	0.059	0.008	0.004	50.883	1.710	0.093	0.187
Hospitalizations	0.211	0.079	0.024	45.187	3.341	0.002	0.008
Disorder duration	0.105	0.079	0.022	37.149	3.594	0.001	0.007
PANSS total	0.038	0.012	0.007	46.750	1.641	0.108	0.188
PANSS positive	0.054	0.066	0.035	47.526	1.912	0.062	0.145
PANSS negative	0.005	0.011	0.019	52.792	0.543	0.590	0.757
PANSS general	0.044	0.026	0.013	40.483	1.950	0.058	0.145
BMI	0.141	0.119	0.033	40.101	3.650	0.001	0.007
CGI	0.005	0.078	0.146	55.704	0.535	0.595	0.757

Abbreviations: CGI, clinical global impression; CPZ, chlorpromazine equivalent; AIM without M, Abstraction, Inhibition and Working Memory test without memory; AIM with M, Abstraction, Inhibition and Working Memory test with memory; VOLT, Visual Objective Learning Test; CVLT, California Verbal Learning Test; BMI, body mass index; PANSS, The Positive and Negative Syndrome Scale; FDR, False-Discovery Corrected. The coefficient of determination for the mixed effect model was calculated according to Jaeger et al. (2016) [19].

Supplementary Table 4. Linear mixed-effect models to explore the associations between BrainAGE and clinical variables

Variable	R^2	Estimate	Std. Error	df	T-value	P-value	P-value (FDR)
CVLT	0.048	-0.107	0.076	31.453	-1.419	0.166	0.212
VOLT	0.040	-0.191	0.145	35.780	-1.315	0.197	0.212
AIM+	<0.001	0.004	0.380	49.923	0.010	0.992	0.992
AIM-	0.033	-0.397	0.299	47.338	-1.328	0.191	0.212
SOFAS	0.041	-0.104	0.073	42.839	-1.426	0.161	0.212
CPZ	0.323	0.104	0.023	40.563	4.526	<0.001	<0.001
Hospitalizations	0.154	0.392	0.140	36.262	2.795	0.008	0.019
Disorder duration	0.219	0.672	0.149	44.698	4.508	<0.001	<0.001
PANSS total	0.200	0.155	0.042	52.850	3.648	0.001	0.002
PANSS positive	0.078	0.452	0.218	52.880	2.078	0.043	0.085
PANSS negative	0.148	0.309	0.107	48.135	2.873	0.006	0.017
PANSS general	0.202	0.316	0.081	47.844	3.893	<0.001	0.001
BMI	0.035	0.320	0.241	51.668	1.326	0.191	0.212
CGI	0.067	1.641	0.871	46.324	1.884	0.066	0.115

Abbreviations: CGI, clinical global impression; CPZ, chlorpromazine equivalent; AIM without M, Abstraction, Inhibition and Working Memory test without memory; AIM with M, Abstraction, Inhibition and Working Memory test with memory; VOLT, Visual Objective Learning Test; CVLT, California Verbal Learning Test; BMI, body mass index; PANSS, The Positive and Negative Syndrome Scale; FDR, False-Discovery Corrected. The coefficient of determination for the mixed effect model was calculated according to Jaeger et al. (2016)[19].

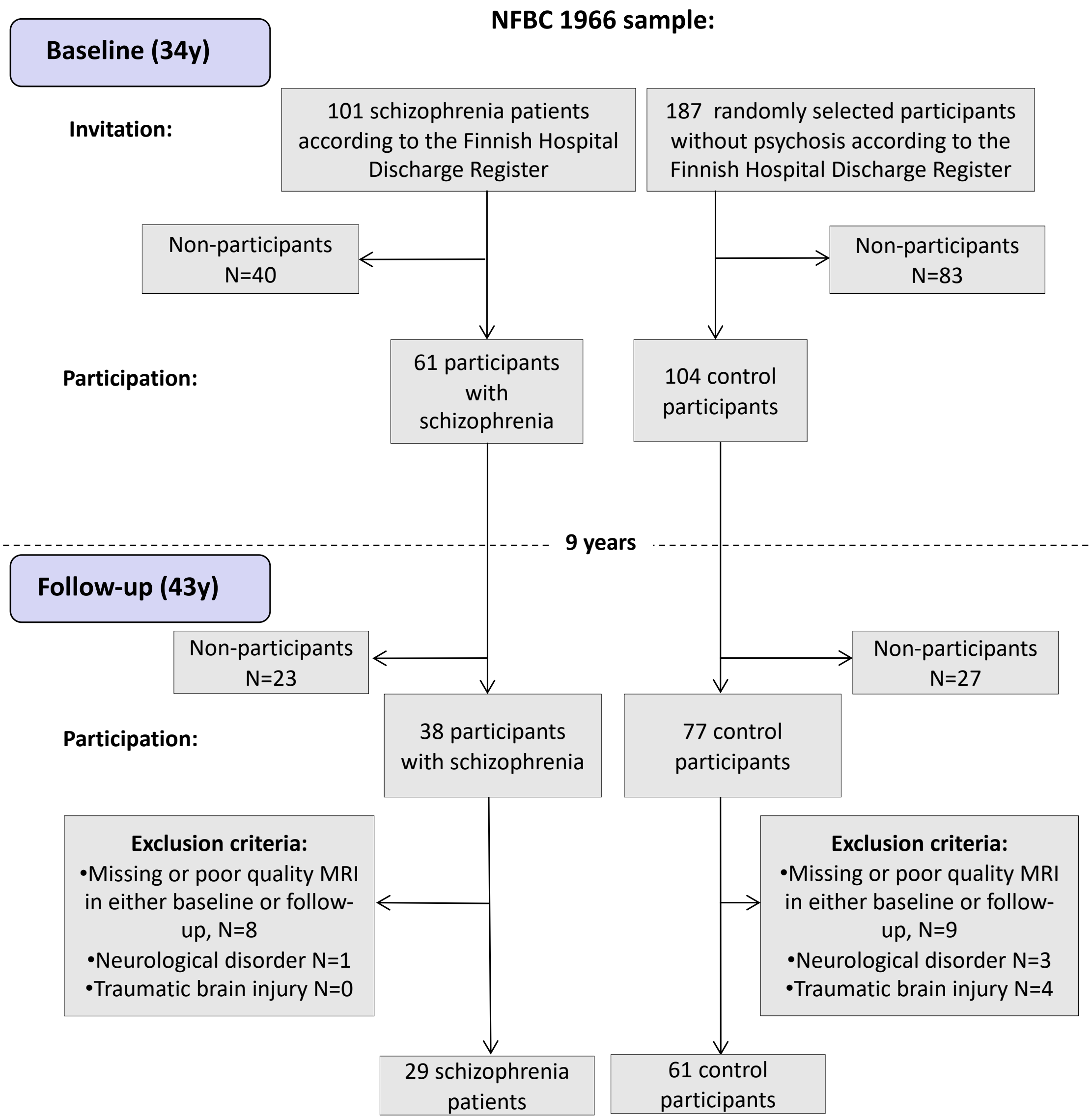

Supplementary Figure 1 The Flow-chart is presenting the NFBC1966 study sample collection for the present study.

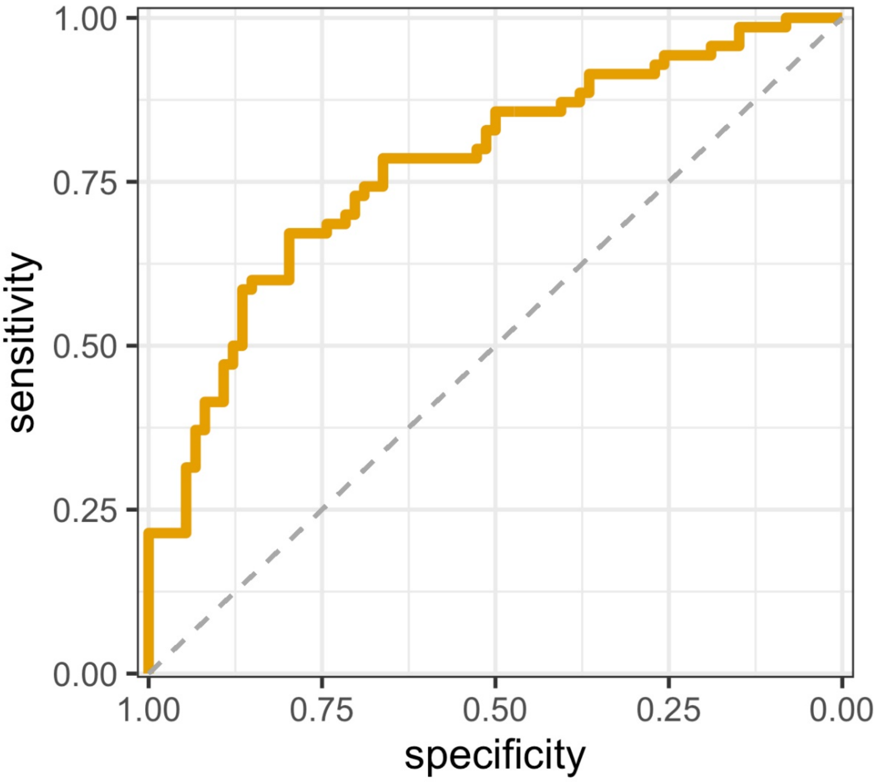

Supplementary Figure 2. The ROC-curve for the COBRE-dataset.

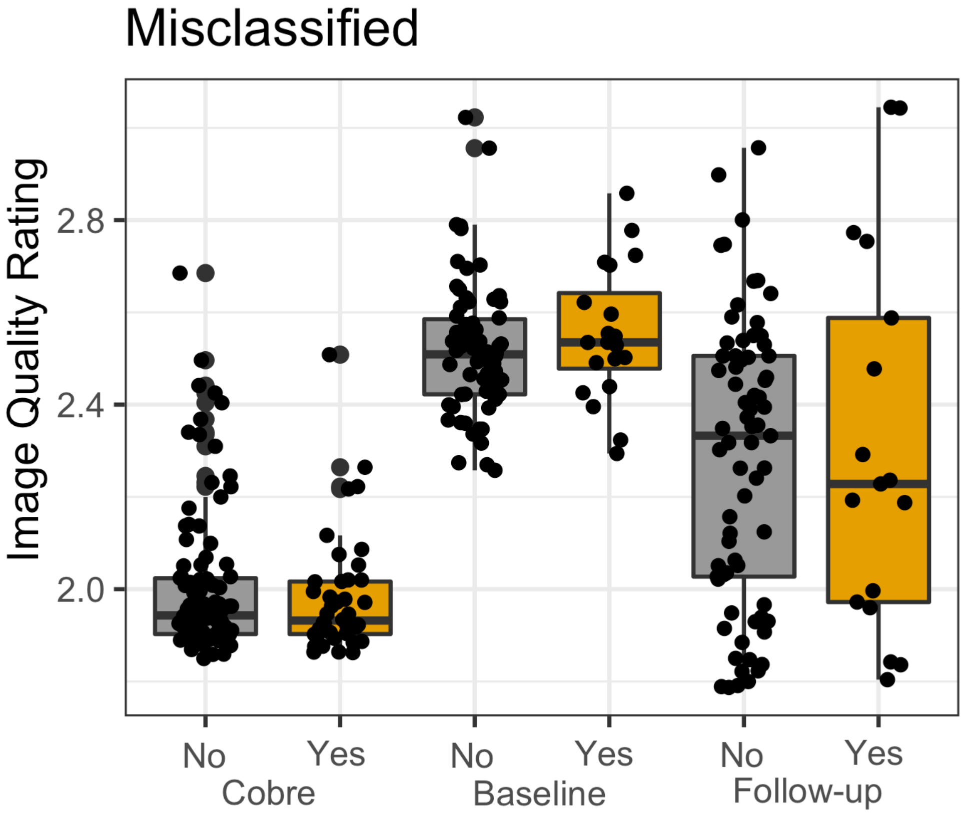

Supplementary Figure 3. The effect of image quality (the lower, the better) on misclassification (Yes/No) at the Cobre sample, and the NFBC1966 (baseline and follow-up). Image quality rating was acquired from the CAT12 output.

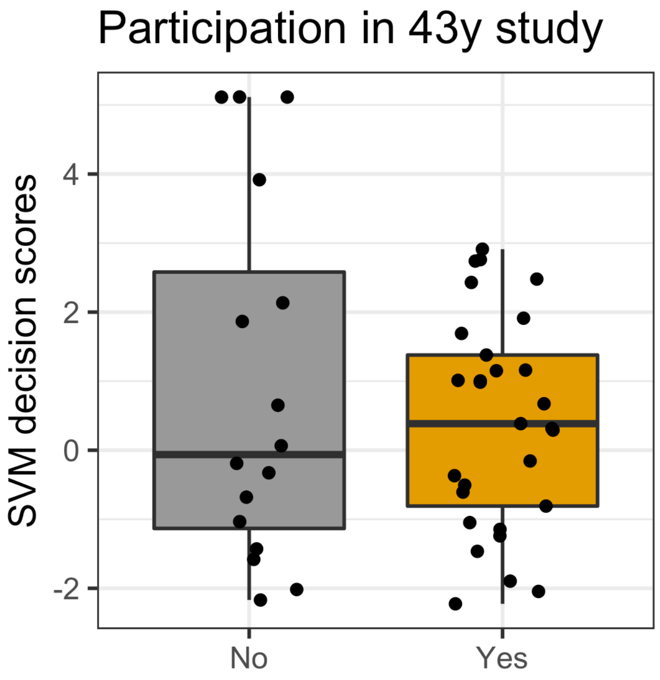

Supplementary Figure 4 The difference in baseline SVM decision scores between those participants who participated in the follow-up (N=29) and those who did not (N=19).

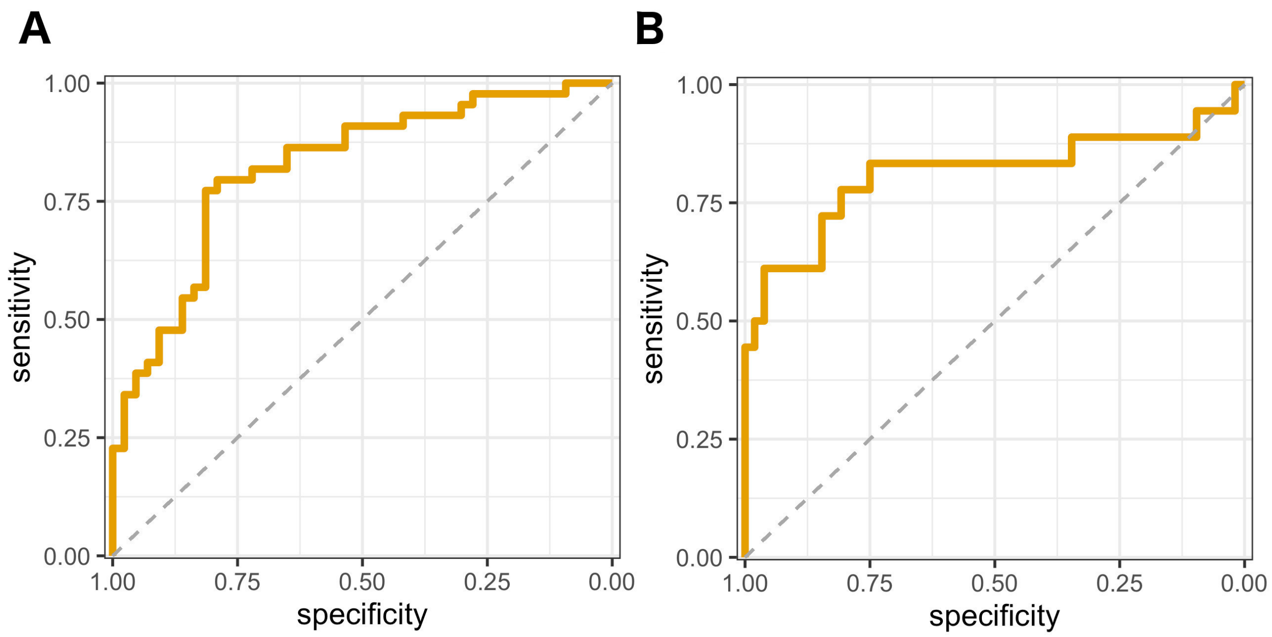

Supplementary Figure 5 ROC-curves for the prediction of schizophrenia in the **a)** NMorphCH and **b)** the CNP samples using the SVM models trained in the COBRE dataset.

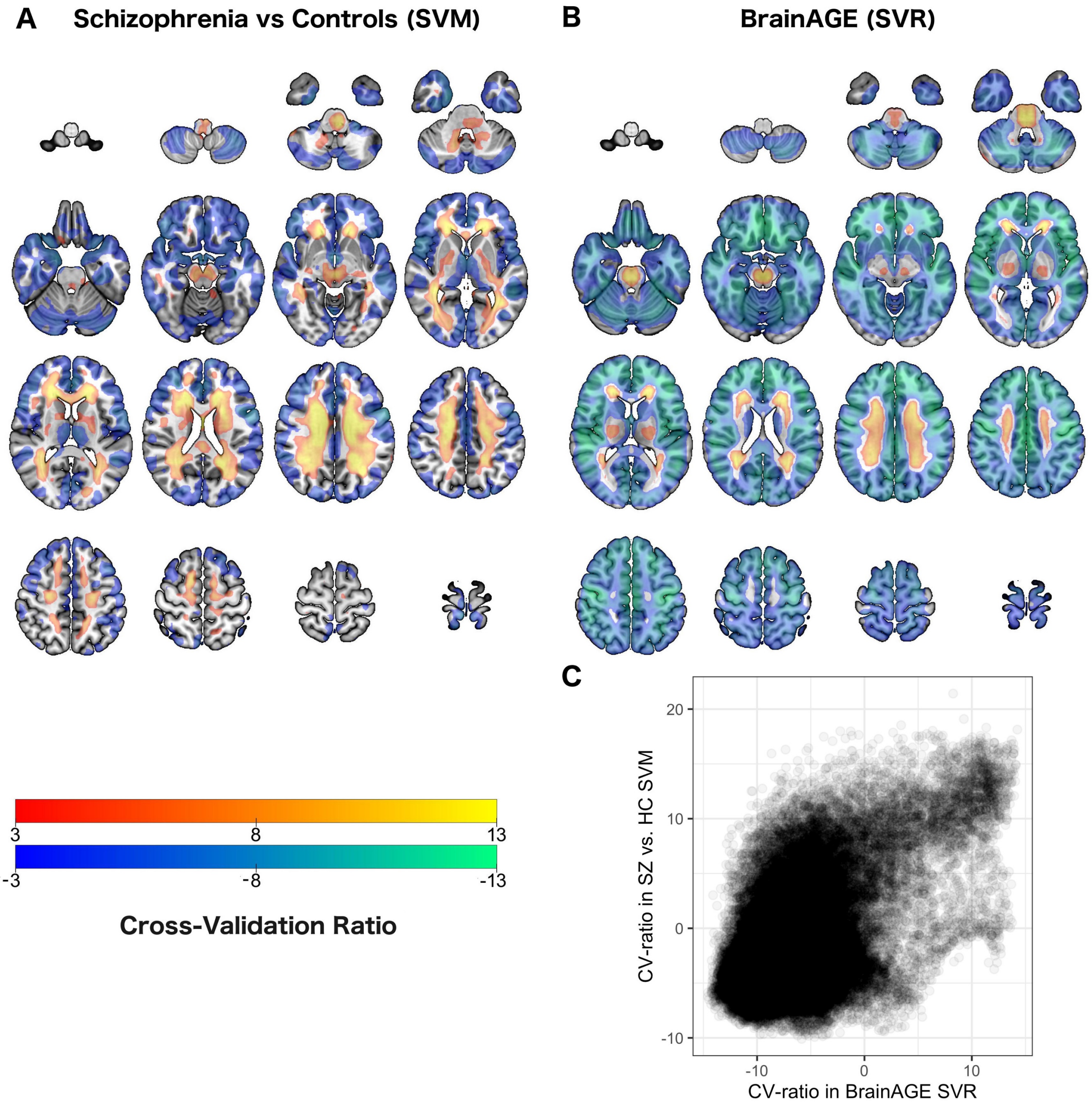

Supplementary Figure 6 a) The Cross-Validation (CV) Ratio in Schizophrenia vs. Controls (SVM). **b)** and BrainAGE (SVR). **c)** The spatial relationship between the CV-Ratios, across all the voxels in the brain, between BrainAGE (SVR) and Schizophrenia vs. Controls (SVM), Spearman's $\rho=0.35$.

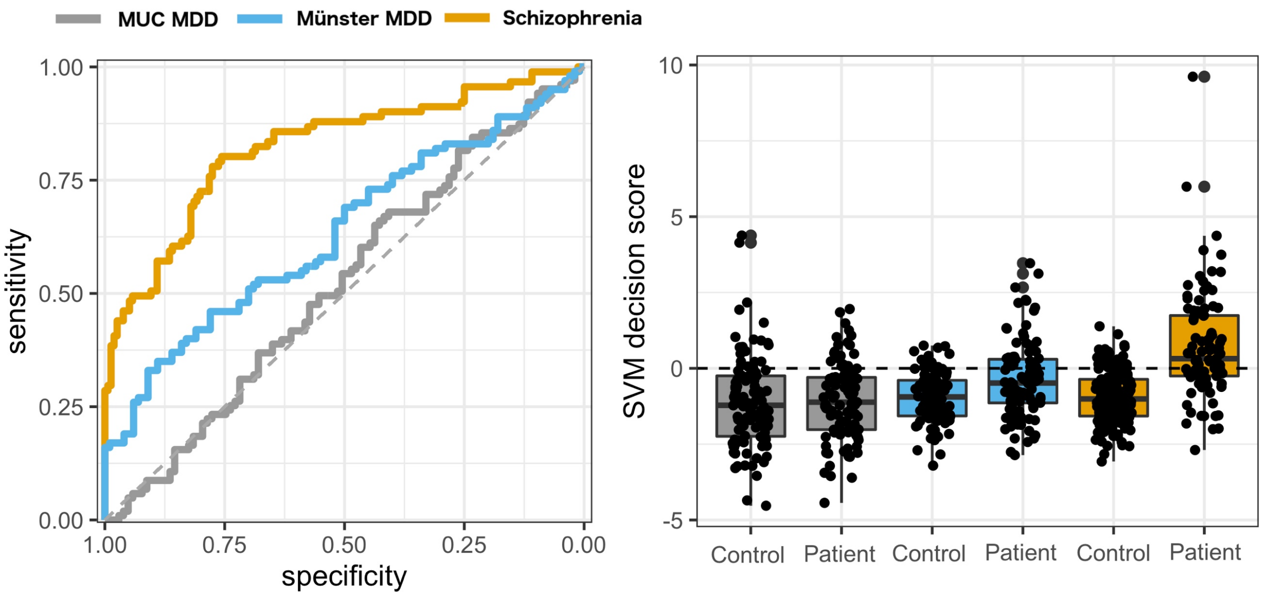

Supplementary Figure 7. The ROC-curves for the prediction of MDD in Munich (103 MDD patients and 103 controls) and Münster (100 MDD and 100 controls) samples, and schizophrenia (91 schizophrenia patients and 156 controls from the NFBC1966, the NMorphCH, and the CNP) using the models trained in the COBRE sample. Boxplot presents the corresponding SVM decision scores. All the predictions were conducted using the COBRE-trained models using OOCV.

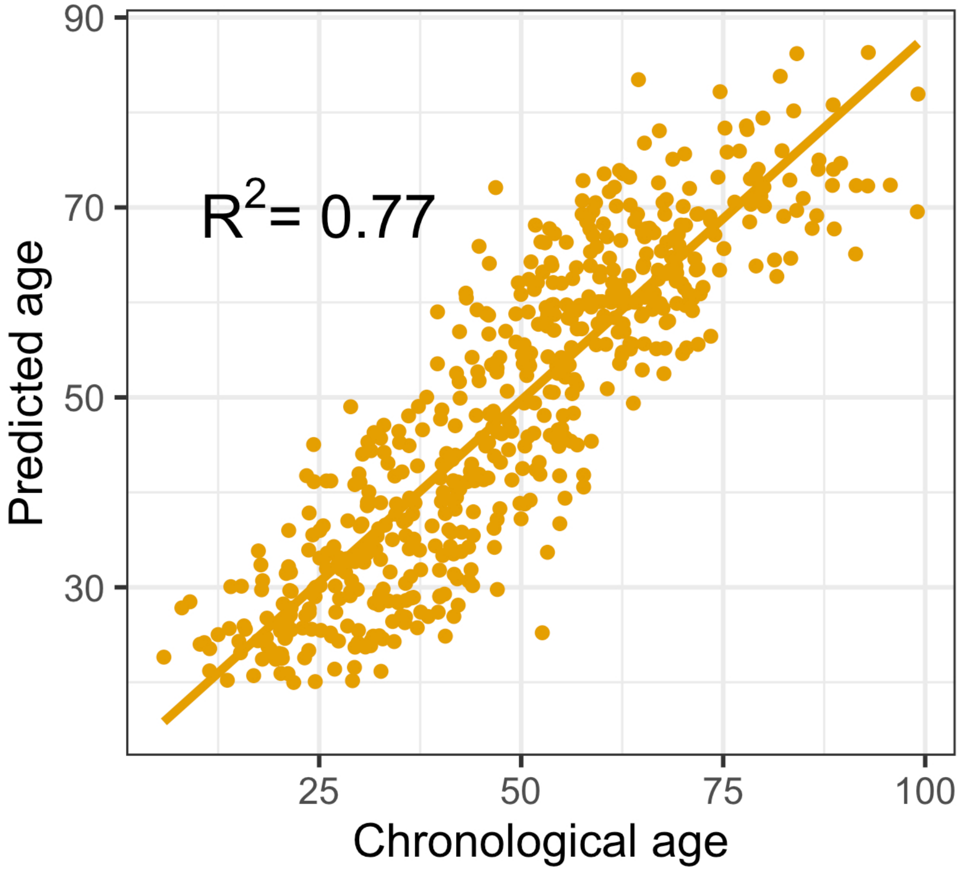

Supplementary Figure 8. Prediction of chronological age from brain images using the IXI sample (N=561).

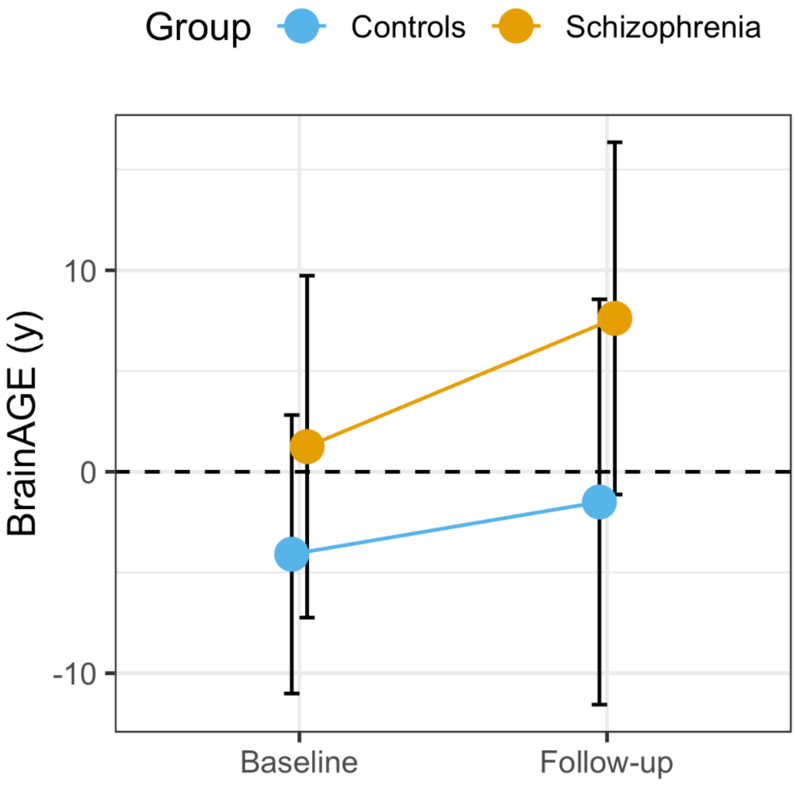

Supplementary Figure 9. BrainAGE at the baseline and the follow-up in the NFBC1966.

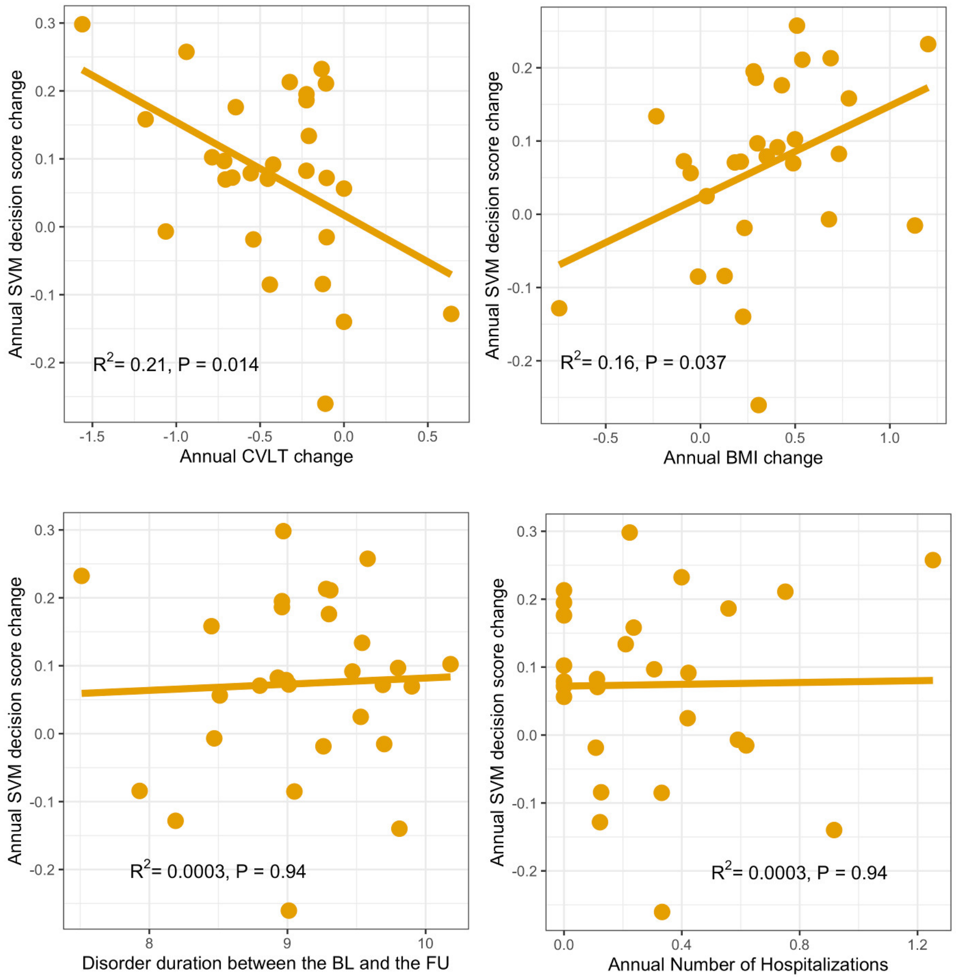

Supplementary Figure 10. Post hoc analyses on the change between SVM decision scores and change in a clinical variable between the baseline and the follow-up. These analyses were restricted to those comparisons where linear mixed models demonstrated a statistically significant relationship.

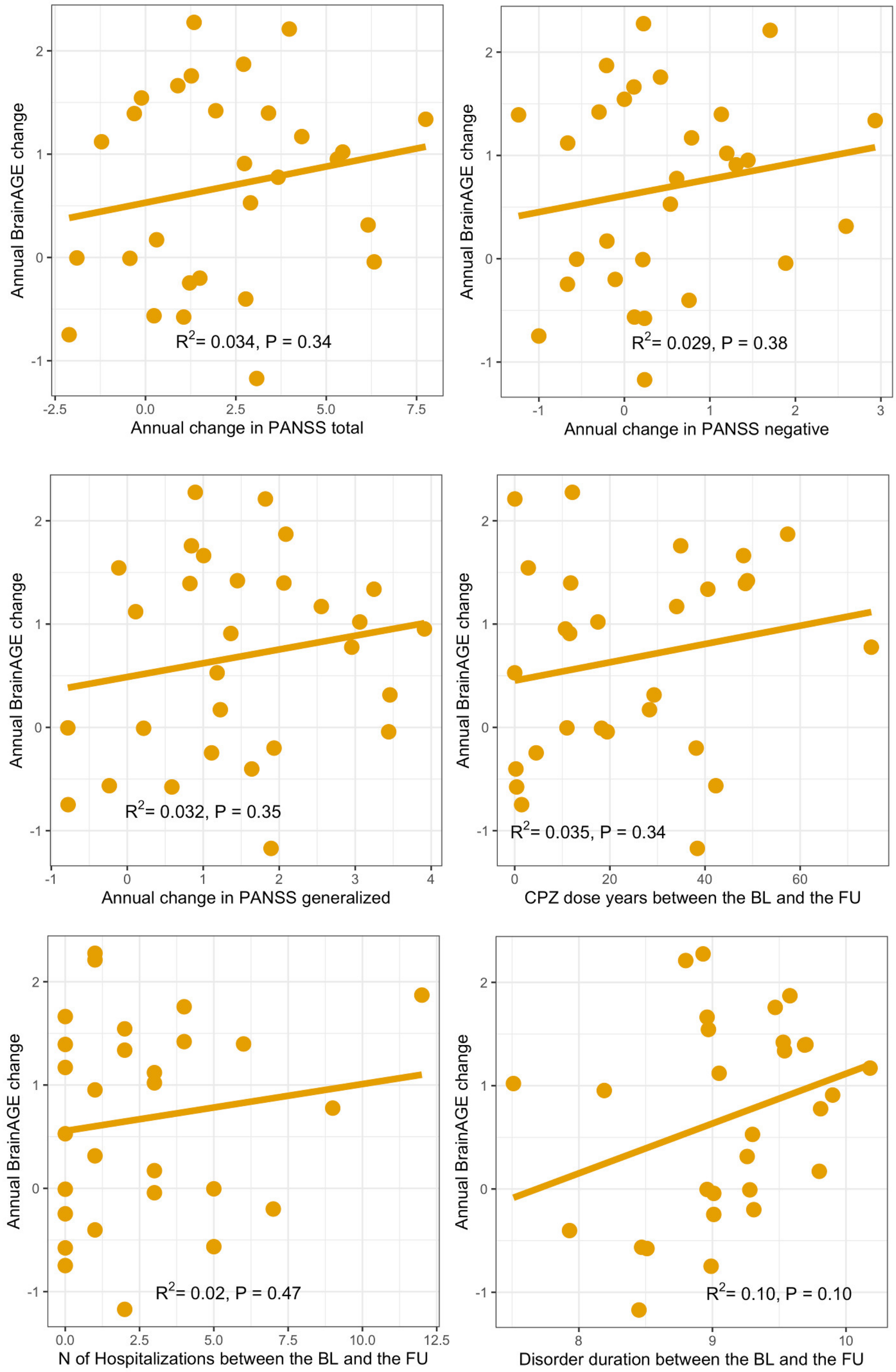

Supplementary Figure 11. Post hoc analyses on the change between BrainAGE and change in clinical variable between the baseline and the follow-up. These analyses were restricted to those comparisons where linear mixed models demonstrated a statistically significant relationship.

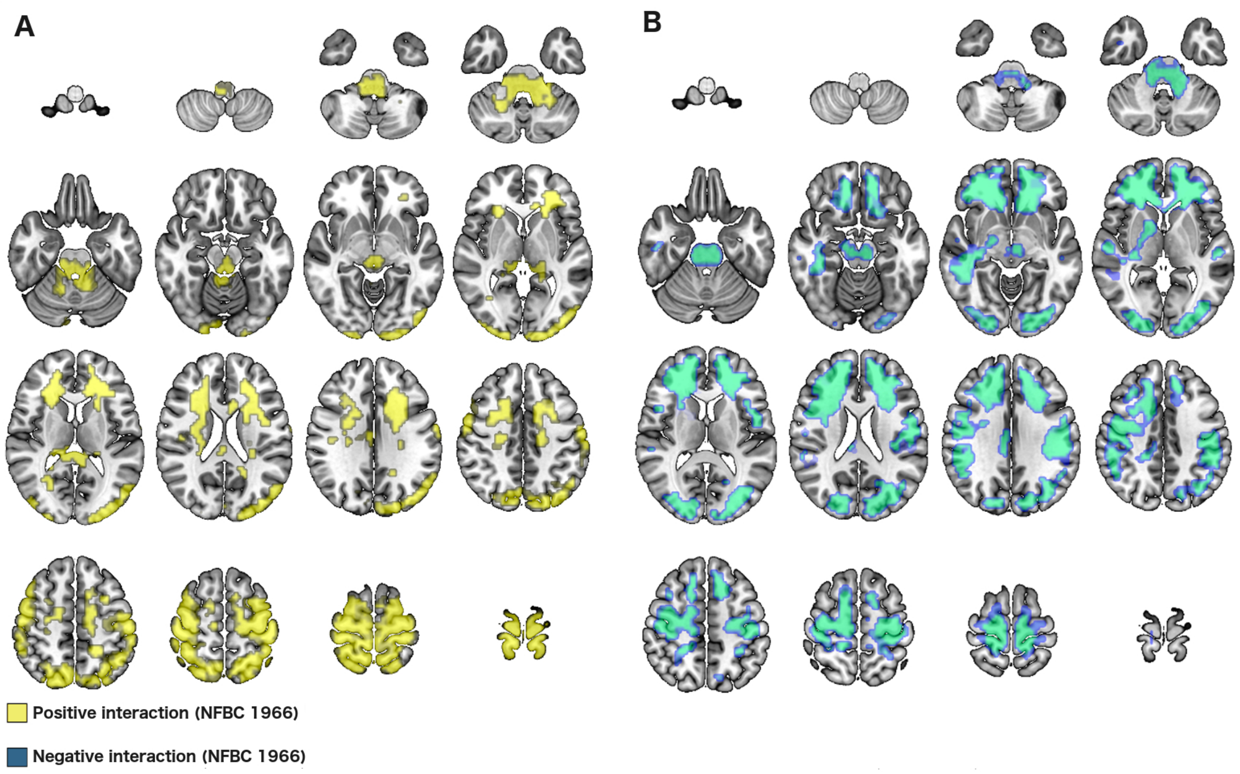

Supplementary Figure 12 a) The positive SVM differences*timepoint interaction on the grey matter density. **b)** The negative SVM differences*timepoint interaction on the white matter density. Note that there were no positive SVM differences*timepoint interaction on the white matter density. Also, we did not find an SVM differences*timepoint interaction on the cerebrospinal fluid density. The segmentations were acquired from the CAT12 output.

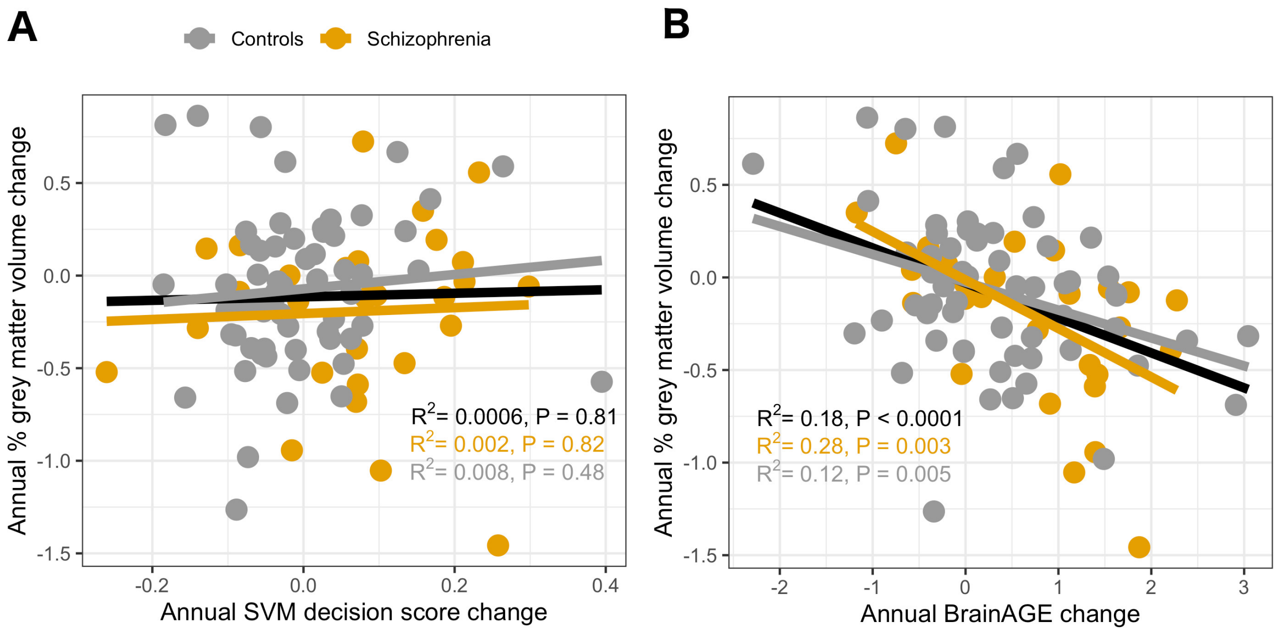

Supplementary Figure 13. a) The relationship between the annual SVM decision score change and annual grey matter volume change. **b)** The relationship between the annual BrainAGE change and annual grey matter volume change. The black line represents the regression slope for the whole sample, yellow for schizophrenia, and grey for controls.

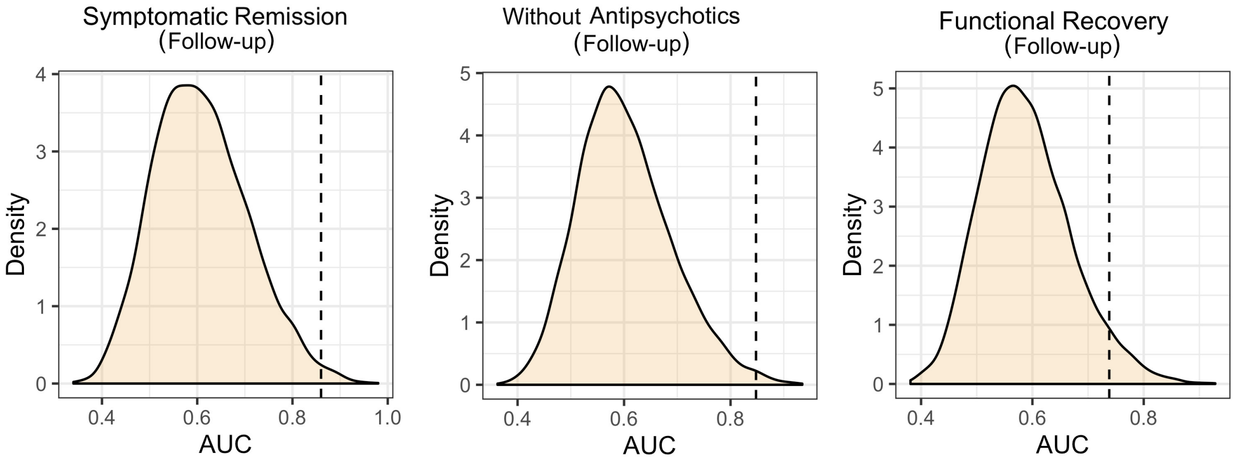

Supplementary Figure 14. Permutation-based tests of the observed area under the curves for predicting future outcomes using SVM decision scores at the baseline (dashed line) compared to an empirical null distribution created by resampling a given future outcome label 5,000 times. Symptomatic remission at the follow-up: uncorrected P-value=0.0116 (FDR-corrected P-value=0.0232). Without antipsychotic medication at the follow-up: uncorrected P-value=0.0066 (FDR-corrected P-value=0.0198). Functional recovery at the follow-up: uncorrected P-value=0.0494 (FDR-corrected P-value=0.0494).

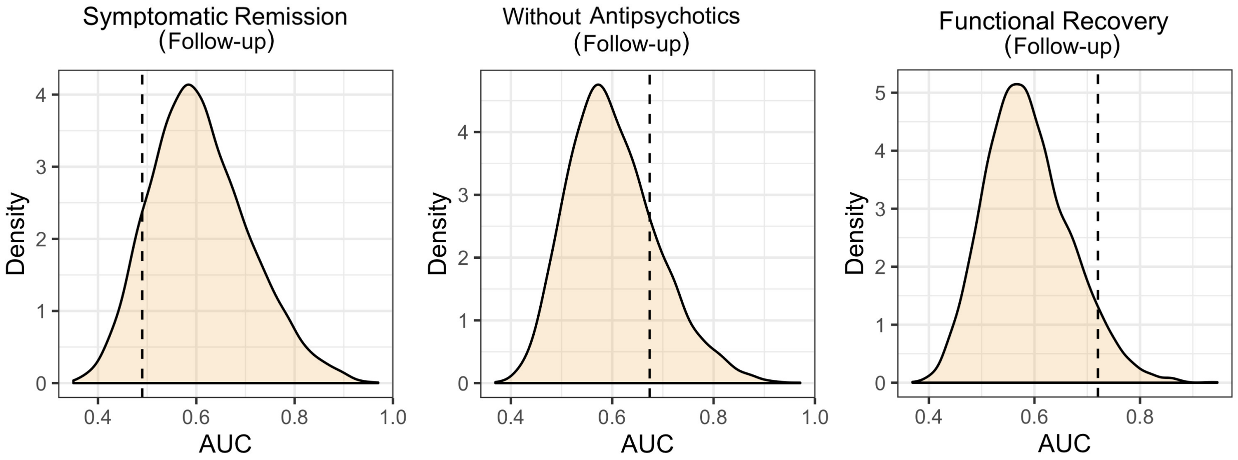

Supplementary Figure 15. Permutation-based tests of the observed area under the curves for predicting future outcomes using BrainAGE at the baseline (dashed line) compared to an empirical null distribution created by resampling a given future outcome label 5,000 times. Symptomatic remission at the follow-up: uncorrected P-value=0.8824 (FDR-corrected P-value= 0.8824). Without antipsychotic medication at the follow-up: uncorrected P-value=0.2098 (FDR-corrected P-value=0.4196). Functional recovery at the follow-up: uncorrected P-value=0.0664 (FDR-corrected P-value=0.1992).

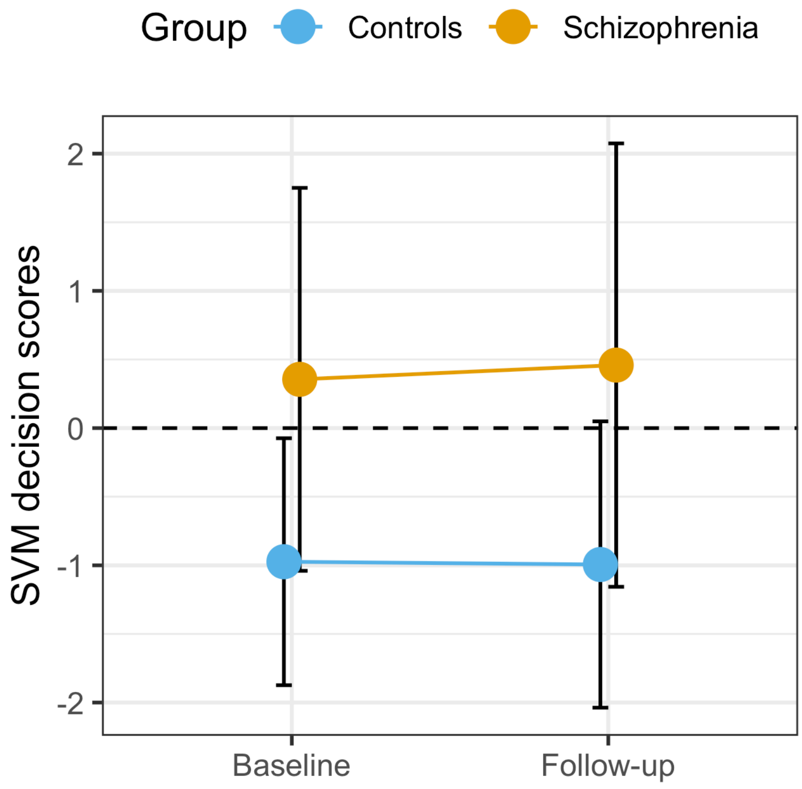

Supplementary Figure 16 The SVM decision scores at the baseline and the follow-up in the NMorphCH (two-year follow-up) using those individuals with all follow-up data.

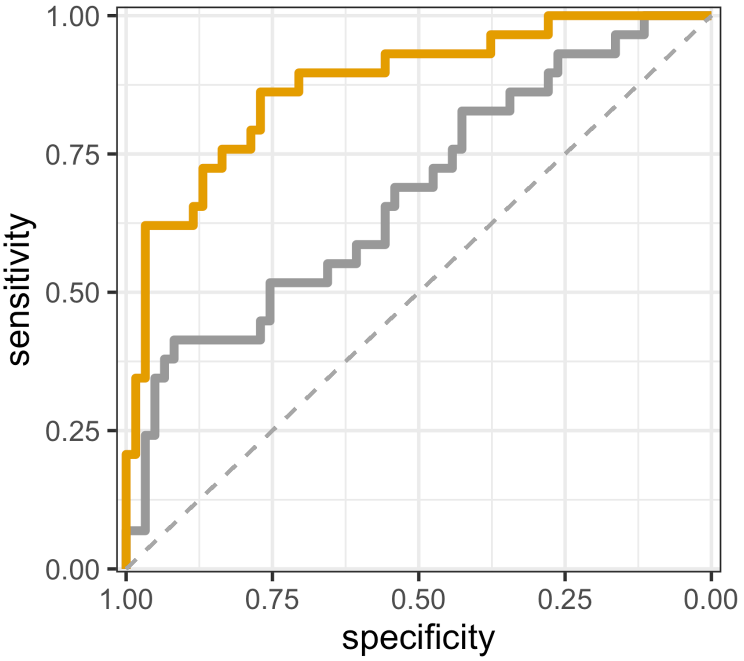

Supplementary Figure 17 ROC-curves for the prediction of schizophrenia at the baseline (grey) and the follow-up (NFBC1966). The models were trained in the NFBC1966 using the same cross-validation design as the main analyses in the COBRE. Grey presents the baseline and yellow the follow-up.

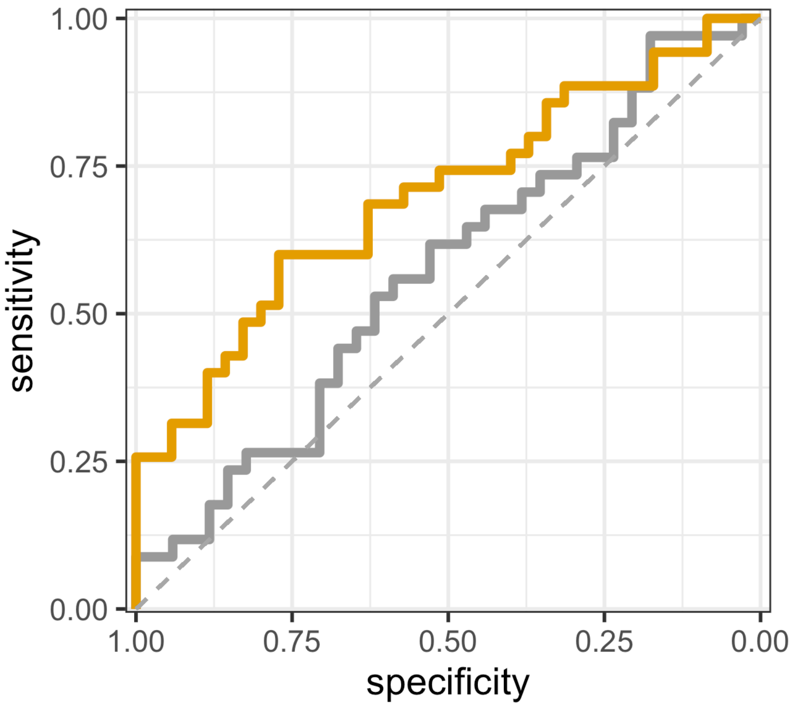

Supplementary Figure 18. ROC curves for the prediction of schizophrenia from controls using the COBRE subsamples. Yellow presents "long" disorder duration subsample and grey "short" disorder duration subsample of the COBRE.

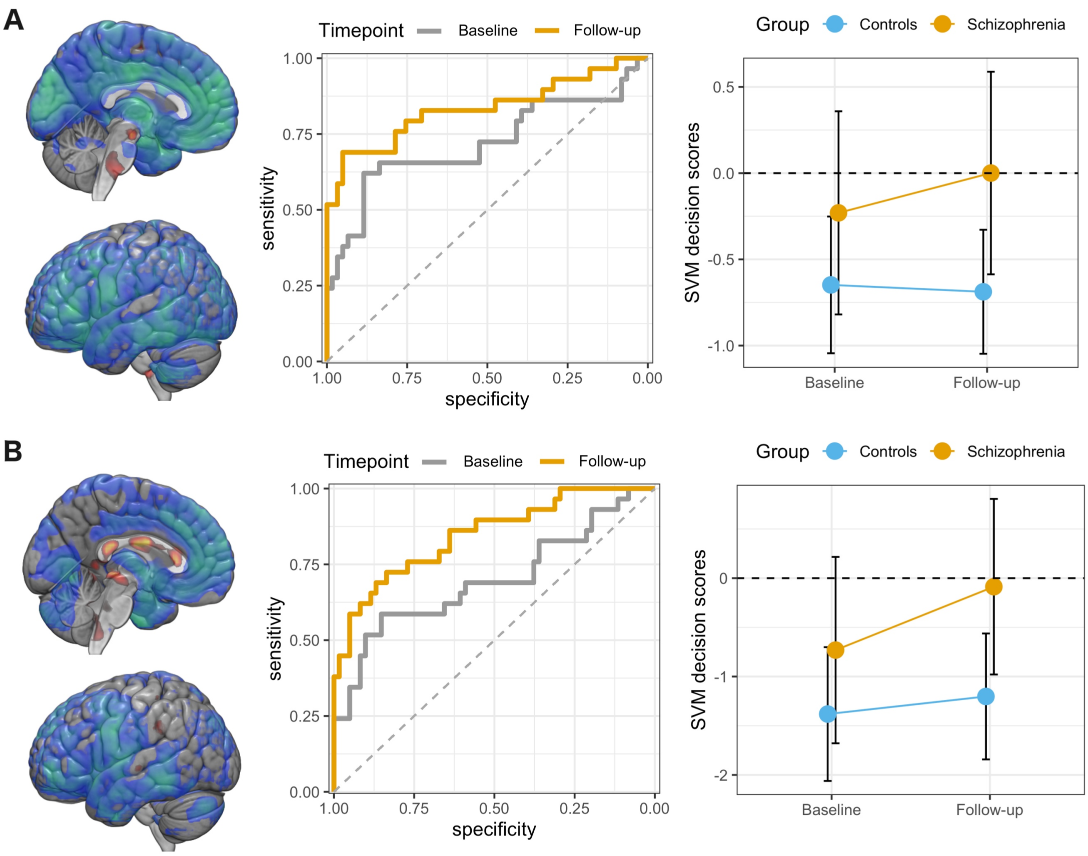

Supplementary Figure 19 a) The prediction of schizophrenia in the NFBC1966 using the "short" disorder duration models trained in the COBRE. **b)** The prediction of schizophrenia in the NFBC1966 using the "long" disorder duration models trained in the COBRE.
