# Supplementary material for: The progression of disorder-specific brain pattern expression in schizophrenia over 9 years
Source: NPJ Schizophr. 2021 Jun 14;7:32. doi: 10.1038/s41537-021-00157-0 (PMC8203625; doi:10.1038/s41537-021-00157-0)
Supplement: Supplementary file 2 — Reporting Summary [file 41537_2021_157_MOESM2_ESM.pdf]

nature research

Corresponding author(s): Johannes Lieslehto, M.D.

Last updated by author(s): 28th of March 2021

Reporting Summary

Nature Research wishes to improve the reproducibility of the work that we publish. This form provides structure for consistency and transparency in reporting. For further information on Nature Research policies, see our Editorial Policies and the Editorial Policy Checklist.

Statistics

For all statistical analyses, confirm that the following items are present in the figure legend, table legend, main text, or Methods section.

n/a	Confirmed	
☐	☒	The exact sample size (*n*) for each experimental group/condition, given as a discrete number and unit of measurement
☐	☒	A statement on whether measurements were taken from distinct samples or whether the same sample was measured repeatedly
☐	☒	The statistical test(s) used AND whether they are one- or two-sided<br>*Only common tests should be described solely by name; describe more complex techniques in the Methods section.*
☐	☒	A description of all covariates tested
☐	☒	A description of any assumptions or corrections, such as tests of normality and adjustment for multiple comparisons
☐	☒	A full description of the statistical parameters including central tendency (e.g. means) or other basic estimates (e.g. regression coefficient) AND variation (e.g. standard deviation) or associated estimates of uncertainty (e.g. confidence intervals)
☐	☒	For null hypothesis testing, the test statistic (e.g. *F*, *t*, *r*) with confidence intervals, effect sizes, degrees of freedom and *P* value noted<br>*Give P values as exact values whenever suitable.*
☒	☐	For Bayesian analysis, information on the choice of priors and Markov chain Monte Carlo settings
☐	☒	For hierarchical and complex designs, identification of the appropriate level for tests and full reporting of outcomes
☐	☒	Estimates of effect sizes (e.g. Cohen's *d*, Pearson's *r*), indicating how they were calculated

Our web collection on statistics for biologists contains articles on many of the points above.

Software and code

Policy information about availability of computer code

Data collection	No software was used in the data collection.
Data analysis	Neuroimaging analyses were conducted using CAT12 and FSL version 6.0.1. Machine learning models were trained using NeuroMiner (version 1.0, https://github.com/neurominer-git/NeuroMiner-1) running on MATLAB r2017a. For statistical analysis, we used the "R" language for statistical computing, version 3.6.3.

For manuscripts utilizing custom algorithms or software that are central to the research but not yet described in published literature, software must be made available to editors and reviewers. We strongly encourage code deposition in a community repository (e.g. GitHub). See the Nature Research guidelines for submitting code & software for further information.

Data

Policy information about availability of data

All manuscripts must include a data availability statement. This statement should provide the following information, where applicable:

- Accession codes, unique identifiers, or web links for publicly available datasets
- A list of figures that have associated raw data
- A description of any restrictions on data availability

The data are not publicly available due to privacy restrictions.

Field-specific reporting

Please select the one below that is the best fit for your research. If you are not sure, read the appropriate sections before making your selection.

☒ Life sciences ☐ Behavioural & social sciences ☐ Ecological, evolutionary & environmental sciences

For a reference copy of the document with all sections, see nature.com/documents/nr-reporting-summary-flat.pdf

Life sciences study design

All studies must disclose on these points even when the disclosure is negative.

Sample size	Given the observational design of the study, no a priori sample size calculation was performed. We conducted several model validations in different samples to assess the generalizability of the models.
Data exclusions	No data were excluded from the study, except extreme outliers (i.e., >3 SD) to meet the normality criteria.
Replication	Schizophrenia vs. Controls classifier's performance was validated in several independent samples.
Randomization	Not applicable. Observational study.
Blinding	Not applicable. Observational study.

Reporting for specific materials, systems and methods

We require information from authors about some types of materials, experimental systems and methods used in many studies. Here, indicate whether each material, system or method listed is relevant to your study. If you are not sure if a list item applies to your research, read the appropriate section before selecting a response.

Materials & experimental systems

n/a	Involved in the study	
☒	☐	Antibodies
☒	☐	Eukaryotic cell lines
☒	☐	Palaeontology and archaeology
☒	☐	Animals and other organisms
☐	☒	Human research participants
☒	☐	Clinical data
☒	☐	Dual use research of concern

Methods

n/a	Involved in the study	
☒	☐	ChIP-seq
☒	☐	Flow cytometry
☐	☒	MRI-based neuroimaging

Human research participants

Policy information about studies involving human research participants

Population characteristics	Multiple datasets including patients with schizophrenia and controls (mean age ~35y).
Recruitment	Multiple recruitment protocols. Please see Supplementary Material for specifics.
Ethics oversight	The local research ethics committees responsible for each dataset approved the present study.

Note that full information on the approval of the study protocol must also be provided in the manuscript.

Magnetic resonance imaging

Experimental design

Design type	Non applicable
Design specifications	Non applicable
Behavioral performance measures	Non applicable

Acquisition

Imaging type(s): Structural

Field strength: 1.5-3 T

Sequence & imaging parameters: Multiple scanning parameters. Please see the Supplementary Material for specifics.

Area of acquisition: Whole brain

Diffusion MRI: ☐ Used ☒ Not used

Preprocessing

Preprocessing software: CAT12, default pipeline.

Normalization: Non-linear standardization.

Normalization template: MNI-152.

Noise and artifact removal: As part of the CAT12 default pipeline.

Volume censoring: Non applicable

Statistical modeling & inference

Model type and settings: Linear Mixed ANOVA

Effect(s) tested: Linear Mixed ANOVA

Specify type of analysis: ☒ Whole brain ☐ ROI-based ☐ Both

Statistic type for inference (See Eklund et al. 2016): Cluster-wise, results reported as T-stats and P-values.

Correction: Permutation.

Models & analysis

n/a	Involved in the study
☒	☐ Functional and/or effective connectivity
☒	☐ Graph analysis
☐	☒ Multivariate modeling or predictive analysis

Multivariate modeling and predictive analysis: The training and testing of the Schizophrenia vs. Controls classifier and brain aging regressor is described in detail in the Methods and Figure 1. Briefly, VBM maps were used as features, PCA was conducted in the cross-validation cycle 1.
